# Genome wide identification and experimental validation of *Pseudomonas aeruginosa* Tat substrates

**DOI:** 10.1038/s41598-018-30393-x

**Published:** 2018-08-09

**Authors:** Maxime Rémi Gimenez, Govind Chandra, Perrine Van Overvelt, Romé Voulhoux, Sophie Bleves, Bérengère Ize

**Affiliations:** 1 Laboratoire d’Ingénierie des Systèmes Macromoléculaires (LISM-UMR7255), Institut de Microbiologie de la Méditerranée, CNRS and Aix-Marseille Univ., 31 Chemin Joseph Aiguier, CS 70071, 13402 Marseille cedex 09, France; 2 Department of Molecular Microbiology, John Innes Centre, Norwich Research Park, Norwich, NR4 7UH UK

## Abstract

In bacteria, the twin-arginine translocation (Tat) pathway allows the export of folded proteins through the inner membrane. Proteins targeted to this system are synthesized with N-terminal signal peptides bearing a conserved twin-arginine motif. The Tat pathway is critical for many bacterial processes including pathogenesis and virulence. However, the full set of Tat substrates is unknown in many bacteria, and the reliability of *in silico* prediction methods largely uncertain. In this work, we performed a combination of *in silico* analysis and experimental validation to identify a core set of Tat substrates in the opportunistic pathogen *Pseudomonas aeruginosa*. *In silico* analysis predicted 44 putative Tat signal peptides in the *P. aeruginosa* PA14 proteome. We developed an improved amidase-based Tat reporter assay to show that 33 of these are real Tat signal peptides. In addition, *in silico* analysis of the full translated genome revealed a Tat candidate with a missassigned start codon. We showed that it is a new periplasmic protein in *P. aeruginosa*. Altogether we discovered and validated 34 Tat substrates. These show little overlap with *Escherichia coli* Tat substrates, and functional analysis points to a general role for the *P. aeruginosa* Tat system in the colonization of environmental niches and pathogenicity.

## Introduction

In Gram negative bacteria, at least 30% of proteins are localized outside the cytoplasm, where they are produced^1^. Bacteria have evolved complex machineries to export these extracytoplasmic proteins across the cellular envelope (inner membrane, periplasm and outer membrane) and secrete them into the extracellular medium or into neighboring target cells^2^. The bacterial cytoplasmic membrane contains two export systems for the translocation of proteins, the Sec and Tat pathways. The Sec pathway is essential and constitutes the major route in most bacteria. Sec-dependent proteins are exported in an unfolded conformation and fold upon delivery to the periplasm. In contrast, the Tat pathway exports proteins in a folded conformation and is used more moderately in most organisms. Tat-dependent substrates are however essential for a wide range of bacterial processes such as adaptation and growth in particular environments, cell division and virulence^3,4^.

Proteins destined to either the Sec or the Tat pathway are synthesized as precursors with N-terminal signal peptides that allow their targeting and recognition by machinery components^5^. Signal peptides have a tripartite organization with a positively charged N-terminal region (N-region), a central hydrophobic core (H-region) and a polar C-terminal region (C-region)^6^. Despite their similar overall organization, Sec and Tat signal peptides have characteristics that allow specific recognition by their respective export pathway. In particular, Tat-dependent signal peptides differ from Sec-dependent signal peptides in the presence of an extended N-region that contains a conserved S/TRRXFLK motif. The two consecutive arginines in this motif are almost always invariant, and gave the system its name^7^. The other amino acids in this motif appear at a frequency greater than 50%^7^. Tat signal peptides are also longer and less hydrophobic than their Sec counterparts^8^. Finally, the C-region of Tat signal peptides contains conserved motifs which are recognition sequences for signal peptidases I and II^8,9^ as well as basic residues that impede targeting to the Sec pathway^10^.

Prediction algorithms that recognize the features of signal peptides based on their amino acid sequence have been developed over the years^11^. SignalP predicts classical Sec signal peptides cleaved by type I signal peptidase. For Tat signal peptides three prediction algorithms are available, TATFIND^12^, TatP^13^ and PRED-TAT^14^. TATFIND searches in the first 35 residues of a protein for the conserved twin-arginines within a specific hydrophobicity context, followed by an uncharged region of a certain position, length and hydrophobicity. TATFIND does not predict cleavage sites. TatP is also based on the search for a specific motif (RR.[FGAVML][LITMVF]), and integrates two machine learning algorithms (Artificial Neural Networks) for cleavage site recognition and hydrophobicity discrimination. Finally PRED-TAT uses a Hidden Markov Model (HMM) method with sub-modules for the distinguishing between Sec, Tat and TM (transmembrane) regions. Comparisons of these prediction algorithms on test sets tend to show that TatP generates fewer false positives than TATFIND and slightly more false negatives^13^. PRED-TAT outperforms both TATFIND and TatP in sensitivity, specificity and in prediction for the precise location of the cleavage site^14^. Overall these algorithms have both strengths and weaknesses, and using them in combination can improve the accuracy of signal peptide prediction. However, parallel experimental confirmation is still required to validate the export pathway and to determine the final localization of a protein. This is particularly true for Tat signal peptides, because the presence of a predicted Tat signal peptide does not always guarantee export via the Tat pathway^15,16^.

One technique used to identify the export pathway of a protein is proteome comparison after cell fractionation of wild type and export defective mutants. Defect in the final localization of a candidate protein in an export mutant can be used to assign the protein to the corresponding export pathway^17^. However the use of such approaches relies on the growth conditions that allow production of the candidate proteins, and on the availability of reliable fractionation procedures. Another means to identify transport pathway dependency is to use transport reporter assays. For Tat dependent transport, assays have been developed that consist in fusing a putative Tat signal peptide to a reporter protein whose Tat-dependent activity can be easily followed upon translocation. Reporter proteins such as the green fluorescent protein^18^, colicin V^19^, maltose binding protein^20^, β-lactamase^21^, agarase^22^ and the N-acetyl muramoyl-L-alanine amidase AmiA^23^ have been used. Of these only the *Streptomyces coelicolor* agarase and *Escherichia coli* AmiA are natural Tat substrates whose export is Tat-specific. Over the years the amidase reporter assay has been used to study the mechanism and function of the Tat system because it offers a facile and sensitive *in vivo* assay for very low levels of Tat activity^24,25^. This assay has also been used in a limited number of cases to confirm the Tat-dependent export of putative Tat substrates^26–29^. However the amidase assay has never been used to screen large numbers of candidate Tat substrates.

*Pseudomonas aeruginosa* is an opportunistic human pathogen, responsible for a variety of chronic and acute infections in susceptible hosts, including immunocompromised patients, those with injury to the epithelial barrier (burns or damages to the cornea) and patients with cystic fibrosis. It is one of the ESKAPE pathogens (*Enterococcus faecium*, *Staphylococcus aureus, Klebsiella pneumoniae*, *Acinetobacter baumannii*, *P. aeruginosa* and *Enterobacter* spp) described as clinically relevant and highly multidrug resistant, and is a major menace to public health worldwide^30^. *P. aeruginosa* virulence is multifactorial and relies on numerous determinants that facilitate bacterial colonization, invasion and dissemination^31^. *P. aeruginosa* has a Tat system with the same TatABC core components as found in *E. coli* where TatC is essential^32^. We and others have shown that the Tat system is essential for full virulence of *P. aeruginosa* and that it plays a broad role in various processes such as the secretion of exoproteins, iron acquisition, anaerobic growth, osmotolerance, motility, biofilm formation, carbon metabolism and detoxification^29,32–36^. Except for a few exceptions, the molecular links between these defective processes and the *tat* mutation are still uncertain. This is mainly because the full repertoire of Tat proteins in *P. aeruginosa* is unknown.

In this study, we used a genome-wide *in silico* approach to identify putative Tat signal peptides in the *P. aeruginosa* PA14 strain. After development of the *E. coli* amidase reporter assay and its validation for *P. aeruginosa* signal peptides, we used it to probe all *P. aeruginosa* candidate Tat signal peptides identified *in silico* and reveal *bona fide* Tat signal peptides. This approach allowed us to confirm the Tat-dependent export of 8 previously known *P. aeruginosa* Tat substrates and to uncover 25 new *P. aeruginosa* proteins with a Tat signal peptide. Our approach also highlighted how the missassignement of one start codon on the PA14 genome impaired the subsequent prediction of a Tat signal peptide on the SphC protein. We then confirmed *in vivo* in *P. aeruginosa* that this signal peptide allows the periplasmic localization of SphC in a Tat-dependent manner. Altogether, our data reveal that *P. aeruginosa* possesses at least 34 Tat substrates and that the Tat system is involved in a wide range of processes allowing adaptation of this opportunistic pathogen to its niche and establishment of host infection.

## Results

### *In silico* screening of the *P. aeruginosa* genome sequence for N-terminal regions encoding twin-arginine signal peptides

**Figure 1:**
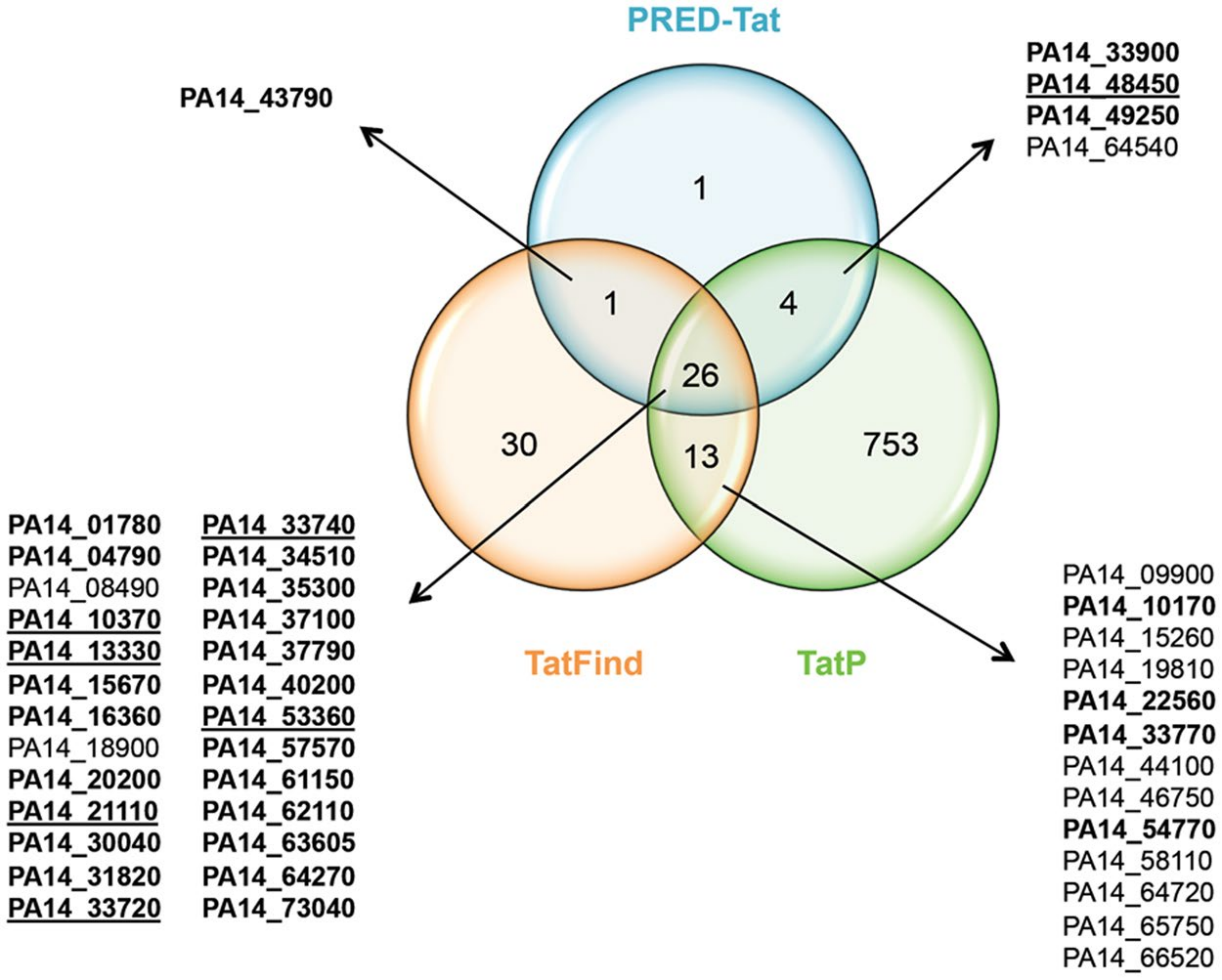
VENN diagram illustrating the comparison between the three Tat prediction program outputs. TATFIND (orange), TatP (green), and PRED-TAT (blue) output of *P. aeruginosa* proteome analysis. The numbers present inside the circles represent the total number of proteins identified with one, two or three prediction programs. Arrows from each overlapping output point to lists of *P. aeruginosa* candidate signal peptides tested in the study. These include 7 already known Tat substrates (underlined): with PA14_53360 (PlcH), PA14_21110 (PlcN), PA14_13330 (EddA), PA14_33740 (PvdP), PA14_33720 (PvdN), PA14_48450 (Agu2A’) and PA14_10370 (PA4140). Signal peptides shown later to target efficiently AmiAH to the Tat pathway (Fig. 6) are indicated in bold.

Genomic and experimental approaches indicate that the Tat system is used to various extents in organisms. While the Tat system is utilized for more than 100 substrates in *Streptomyces* species, it is used for 27 substrates in *E. coli*, and only for 5 to 7 substrates in *Bacillus subtilis* despite the presence of two independent Tat machineries^37^. The *P. aeruginosa* proteome has been manually screened for the presence of Tat signal peptides in two studies either looking for the motif RRXFLK/R^33^ or the two conserved arginines followed by at least two of the FLK/R residues^37,38^. These studies respectively led to the prediction of 18 and 27 putative Tat signal peptides with 10 signal peptides common between them. Here, we screened the *P. aeruginosa* PA14 proteome for Tat signal peptides using two Tat prediction programs, TATFIND and TatP. Although the twin-arginine motif is highly conserved in Tat substrates, natural non canonical Tat signal peptides exist, where one of the consensus arginine residues is replaced by a lysine^39,40^. To take into account exceptions that might also exist in *P. aeruginosa* we modified the TATFIND and TatP prediction algorithm to consider RK or KR the same as RR. This analysis led to the prediction of 72 putative Tat signal peptides by TATFIND and 794 by TatP respectively (Table S1). As expected from the conceptual differences between these algorithms the candidate Tat signal peptides found show partial overlap and TatP predicted considerably more candidates than TATFIND. We selected the 39 Tat signal peptides predicted by both algorithms for further analysis (Table 1A and Fig. 1). To avoid dismissing real Tat substrates predicted only by TATFIND or TatP as occurred in *Streptomyces coelicolor*^22^, we next compared our list of candidates with the candidates obtained with the most recent program, PRED-TAT. This allowed us to select another five candidate signal peptides that were positive with this program (and either TatP or TATFIND) for further analysis (Table 1B and Fig. 1). The final set contained 44 putative Tat signal peptides. Of these, seven out of the eight already known *P. aeruginosa* Tat signal peptides (PA14_53360/PlcH, PA14_21110/PlcN, PA14_13330/EddA, PA14_33740/PvdP, PA14_33720/PvdN, PA14_48450/Agu2A’and PA14_10370/PA4140) were present (Fig. 1). Indeed, up to now 8 Tat substrates have been experimentally identified in *P. aeruginosa*: PlcH and PlcN^32^, PvdN^34^, PvdP^35^, Agu2A’^36^ and EddA, PA2699/PA14_29230 and PA4140^29^. It was not surprising that the PA2699 Tat signal peptide was not identified in our analysis, because its start codon is missassigned on the *P. aeruginosa* genome^29^. While the Tat signal peptides of PlcH, PlcN, EddA, PvdP, PvdN, and PA4140 were identified by the three prediction programs, the Agu2A’ signal peptide was identified only by TatP and PRED-TAT. Among the final set 40 putative Tat signal peptides contained the typical twin-arginine repeat, while two possessed an RK (PA14_46750 and PA14_64720) and two a KR (PA14_44100 and PA14_09900). Altogether our *in silico* analysis identified 44 putative Tat signal peptides in *P. aeruginosa* PA14. 37 of these have never been experimentally tested for their dependence on the Tat system.

**Table 1:** Putative Tat signal peptides of *P. aeruginosa* identified *in silico*.

**PA14 Gene id**	**PAO1 Gene id**	**Sequence from N-terminus** ^**a**^
**A. Tat signal peptides predicted by TATFIND and TatP**		
PA14_01780	PA0144	MSRSNGSSS**RR**T**FL**RLAALLLPAGALLGSLPGVRA*
PA14_04790	PA0365	MSDTTLESAGL**SRR**SLM**K**VGLIGGAFLATA*
PA14_08490	PA0663	MSGWELQFRDP**RR**AWLVRLGVGALLLAVPLAFLGGRWSTGADA*
PA14_09900	PA4175^b^	MH**KR**TY**L**NA**C**LVLALAAGASQASA*
PA14_10170	PA4159	MP**TRR**RSALPLLALALSLFATLAAA*
PA14_10370	PA4140^d^	MHDPIQQADAFVDDPDQESGGL**SRR**S**FL**GKSATLGAVGLVAGWTPAFVIQPAEA*
PA14_13330	PA3910^d^	MSGMDLK**RR**RVVQGLGAGLLLPALGAPAVIA*
PA14_15260	PA3774	M**TRR**TAFFFDELCLWHAAGPHALTLPVGGWVQPPAAAGHA*
PA14_15670	PA3768^c^	MTF**TRR**QV**L**GGLAGLAVVGLGAGG*ARLWLARPQVA*
PA14_16360	PA3713	MTI**SRR**D**FL**NGVALTIAAGLTPAEILRA*
PA14_18900	PA3493	MDAA**TRR**SM**L**RNALLLGLFALVGVGLVALV*
PA14_19810	PA3422	MTTTK**RR**GIFPLHALAAATALG**C**ATQASA*
PA14_20200	PA3392	MSDDTKSPHEETHGLN**RR**G**F**LGASALTGAAALVGASALGSAVVGREARA*
PA14_21110	PA3319^d^	MISK**SRR**S**F**IRLAAGTVGATVATSMLPSSIQA*
PA14_22560	PA3222	MN**RR**SA**L**AALHGGALLFGLTGVFGKLASA*
PA14_30040	PA2635	MSLEKKDAILFGDGDELPSNHSNNPHMNDLIAGLG**RR**Q8V**L**AGGAALGALAFLGVALPASA*
PA14_31820	PA2531	MPAL**SRR**S**F**VTLTALASSSILLSPRAFA*
PA14_33720	PA2394^d^	MND**RR**T**FLK**QAGILAAGLPLLSAAQSLRA*
PA14_33740	PA2392^d^	MTV**SRR**G**F**MAGLALTGAAALPVA*
PA14_33770	PA2389	MRRTRS**TRR**AL**L**VAVCLSPLIALA*
PA14_34510	PA2328	MCLDDPTH**SRR**DI**LK**LAALLSAAGALPLLSSLQARA*
PA14_35300	PA2264	MPDDKAVNG**RR**D**FL**RKTLTVIPAVTLAGYGVGHAMQTPA*
PA14_37100	PA2124	MHQPENPA**RR**TL**L**AQTVAGSAALALGSLLGGAPGVASA*
PA14_37790	PA2065	MHRT**SRR**T**F**V**K**GLAATGLLGGLGLWRAPAWA*
PA14_40200	PA1880	MNSKIDLSNALPG**SRR**G**FLK**GAAVVGLTIGFQWSGARRALA*
PA14_44100	PA1578^b^	MRLH**KR**SLVWGLALSGLAVVLAAAWWASQA*
PA14_46750	PA1356^b^	MSERLYVGT**RK**GLFELRRNAAGQWLPMASHFLG*
PA14_53360	PA0844^d^	MTENWKFR**RR**T**FLK**HGAQAATLAGLSGLFPETLRRALA*
PA14_54770	PA0735	MNRN**RR**NALIAGSLLLLAANLAALGGVAWNRS*
PA14_57570	PA4431	MSNDGVNAG**RR**R**FL**VAATSVVGAAGAVGAAVPFVGSWFPSAKAKA*
PA14_58110	PA4478	MPSLYLASASPR**RR**EL**L**TQIGVPLSVLATAIDESPLPNEAPA*
PA14_61150	PA4621	MSNRDI**SRR**A**FL**QGGLIAGVGVTLAPLGSQAFA*
PA14_62110	PA4692	MLIKIPSRSDCSESE**V**TSETLYL**SRR**RL**L**GASFAGLALASGLPRLGFADE*
PA14_63605	PA4812	MDMN**RR**Q**F**F**K**VCGIGLGGSSLAALGMAPTEAFA*
PA14_64270	PA4858	MK**RR**SL**LK**AFTLSASIASMGLSWSIQA*
PA14_64720	PA4898^b^	MKIR**RK**TAIPRNAGAVLPMLACLAAQA*
PA14_65750	PA4974	ML**RR**LS**L**AAAVAAATGVAWA*
PA14_66520	PA5031	MHPWA**RR**NIPQLAGRLALVTGANSGLGWQAARTLA*
PA14_73040	PA5538	MK**RR**RL**L**QSLLAGLALQPFLAASA*
**B. Tat signal peptides predicted by TATFIND or TatP and PRED-Tat**		
PA14_33900	PA2378	MKRSFPDDLVIGNL**SRR**G**FLK**GVGATGVLLVAANWGWRDALA*
PA14_43790	PA1601	MSLANP**SRR**G**FLK**AGGLLLVTVNLPAPLLALA*
PA14_48450^d^	Not_conserved	M**TRR**H**FL**QRLGASAGLGAALTLGLEFGSPRGQA*
PA14_49250	PA1174	MNL**TRR**E**F**A**K**ANAAAIAAAAAGLPILVRA*
PA14_64540	PA4882	MKGPEKKRAKIAIDPSSERQMVDLQ**RR**LL**L**RGGLSLGALAMLSGCRLQ*

^a^The putative twin arginine motif is indicated in bold (based on the S/TRRxFLK motif), the hydrophobic domain underlined and the cleavage site indicated by an asterisk.

^b^The putative twin arginine is degenerated in RK or KR.

^c^Two versions of this signal peptide (designated AGG and QVA) with two alternative leader peptidase cleavage sites were tested (see Fig. 5).

^d^Already known Tat substrates.

### Development of the amidase Tat reporter assay

**Figure 2:**
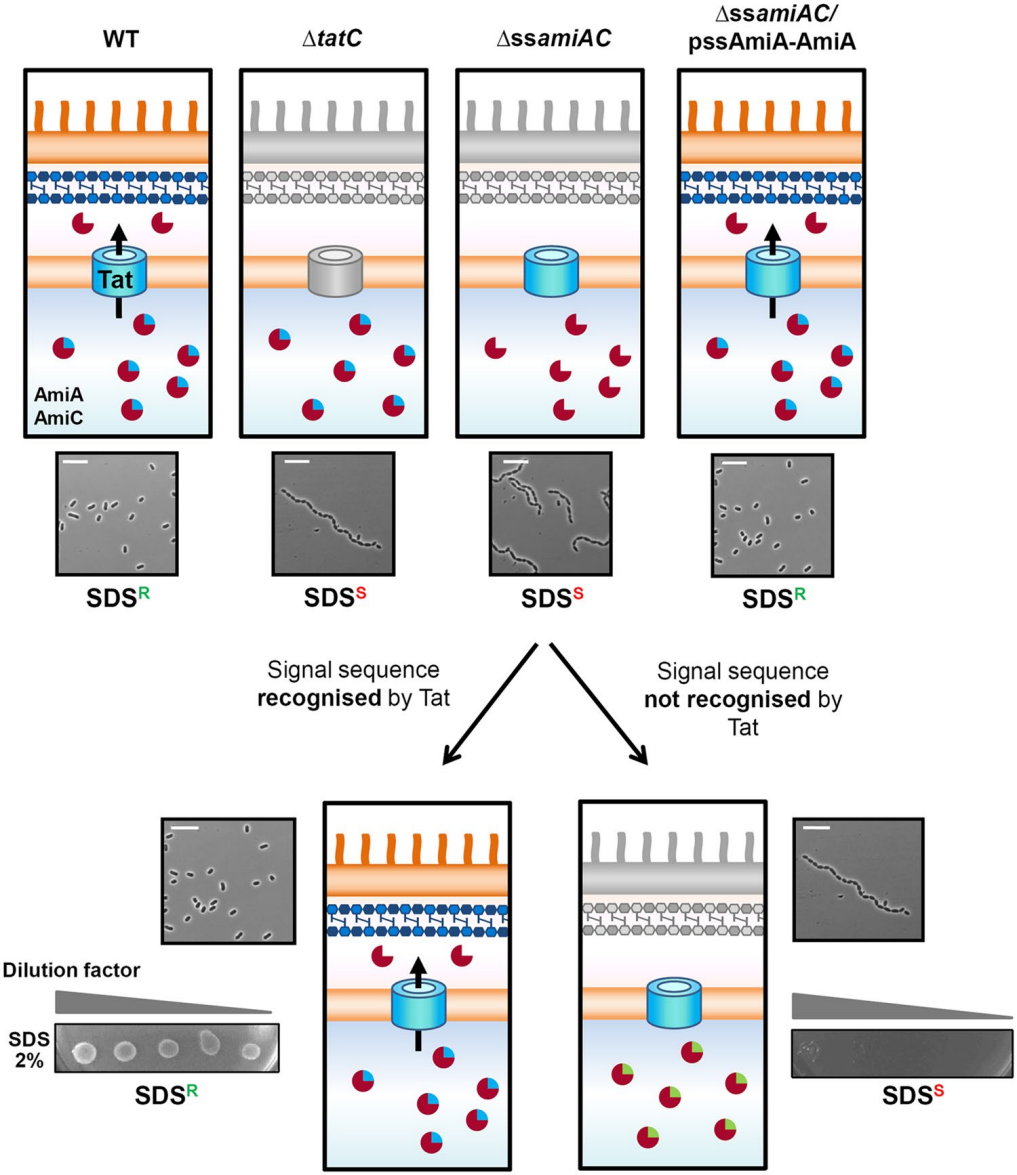
Schematic representation of the amidase reporter assay or SDS sensitivity: an easy genetic screen to probe Tat signal peptides. In *E. coli* two Tat-dependent amidases, AmiA and AmiC allow the correct cleavage of the peptidoglycan (PG) layer in the periplasm. In the Δ*tatC* mutant (B1Lk0), the absence of export of AmiA and AmiC leads to a defect in cell division and envelope integrity (schematized here by a grey outer membrane). Consequently the Δ*tatC* mutant forms chains of cells and is sensitive to detergent. A Δ*ssamiAC* strain (MCDSSAC), where AmiA and AmiC signal sequences have been deleted shows identical phenotypes to the *tatC* mutant^41^. Only providing AmiA *in trans* in this strain (Δ*ssamiAC* pssAmiA-AmiA) allows the restoration of both cell division and SDS resistance in a Tat dependent manner. The amidase assay has been designed around the *E. coli* MCDSSAC strain and around the fact that AmiA alone is able to restore the envelope defect of the strain. In this assay, if the AmiA signal peptide of pssAmiA-AmiA is replaced by a signal peptide which can be recognized by the Tat system, SDS sensitivity and chain formation can be restored, unlike when AmiA signal peptide is swapped for a sequence not recognized by the Tat machinery. In microscopic images, the white bar represents 10 μm.

To experimentally validate selected putative *P. aeruginosa* Tat signal peptides, we next used the *in vivo* amidase reporter assay that we participated in developing for *E. coli* Tat candidates (ref.^23^ and Fig. 2). This assay indicates whether signal peptides of candidate Tat substrates can engage with the Tat pathway and permit translocation. It uses an *E. coli* strain where the Tat signal sequences of AmiA and AmiC, two Tat-dependent N-acetylmuramoyl-L-alanine amidases, have been deleted (Δ*ssamiAC*^41^). AmiA and AmiC hydrolyse the peptide moiety from N-acetylmuramic acid in peptidoglycan during cell wall biogenesis. Δ*ssamiAC* forms chains of cells and cannot grow in the presence of SDS because the peptidoglycan barrier is damaged. Notably these two phenotypes can be restored in a strictly Tat-dependent manner by AmiA sole production *in trans*. In the amidase-reporter assay a candidate signal peptide is fused to the mature AmiA coding sequence in place of the cognate Tat signal peptide and the chimeric protein is produced in the Δ*ssamiAC* mutant. True Tat signal peptides allow the Tat-dependent translocation of AmiA and restore the ability of Δ*ssamiAC* to form single cells and to grow in the presence of SDS. If the Tat signal peptide candidate is not functional AmiA is not exported and the Δ*ssamiAC* mutant still forms chains of cells and does not grow in the presence of SDS (Fig. 2). This system has been used to show the Tat-dependence of single substrates with signal sequences from *Salmonella enterica* serovar Typhimurium^28^, *Archaeoglobus fulgidus*^26^ and *Streptomyces coelicolor*^27^. More recently we used this system to confirm proteomic data indicating that PA2699 was a genuine extracellular Tat substrate in *P. aeruginosa*^29^. However, this assay has never been used to systemically screen candidate Tat substrates.

**Figure 3:**
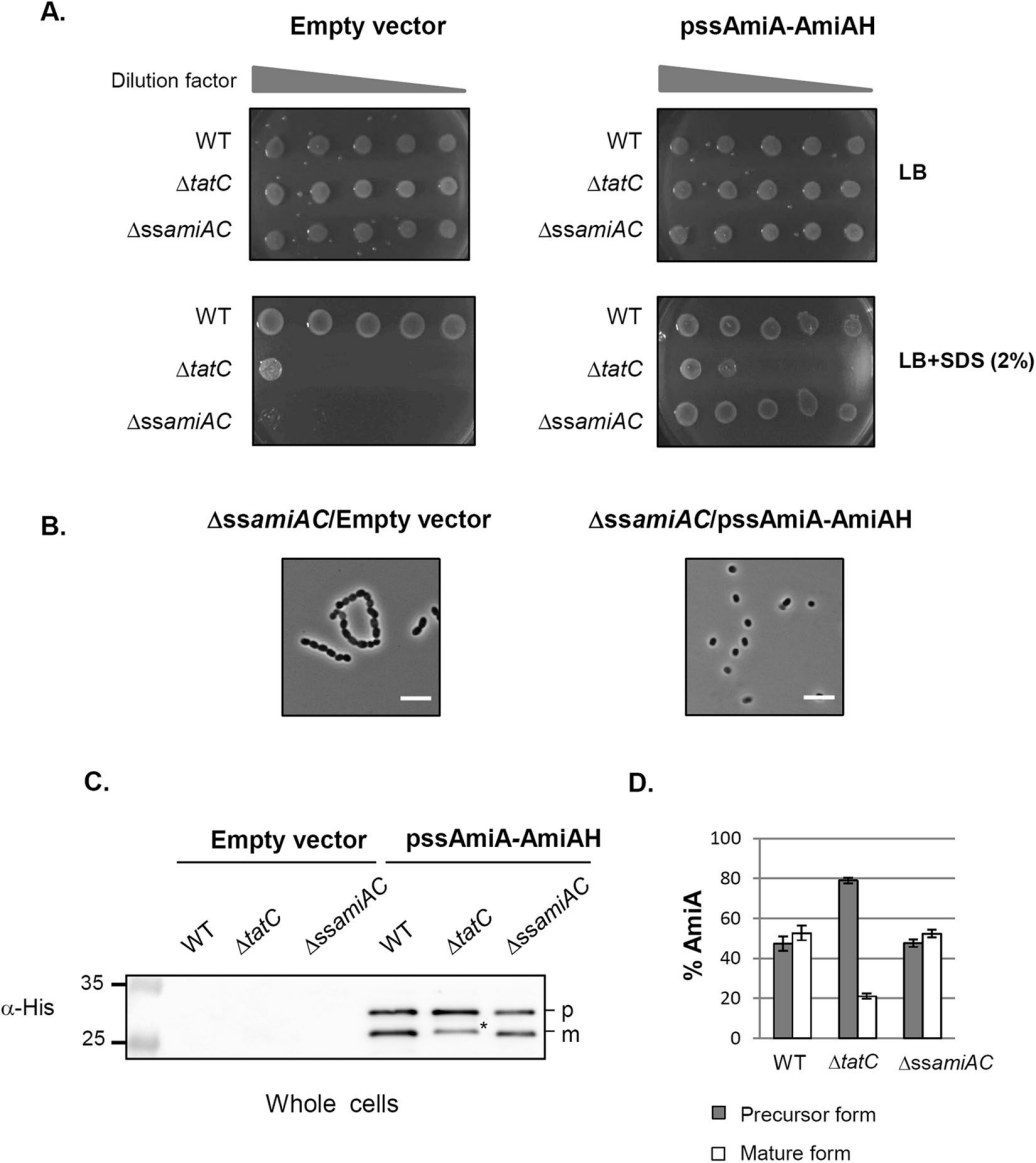
The ssAmiA-AmiAH protein fusion is functional. (**A**) SDS viability assay of wild type (MC4100), Δ*tatC* (B1LK0) and Δ*ssamiAC* (MCDSSAC) strains carrying the empty vector (pUNIPROM) or pssAmiA-AmiAH (producing *E. coli* AmiA with an His_6_ epitope-tag). Strains were grown aerobically and 5 μl were spotted on LB agar plate containing 2% (w/v) SDS as indicated in the material and methods section (**B**) Microscopic observation of Δ*ssamiAC* strains carrying the empty vector or pssAmiA-AmiAH. Scale: the white bar represents 10 μm (**C**) Immunoblot analysis of whole cells prepared from wild type, Δ*tatC* and Δ*ssamiAC* strains carrying the empty vector or pssAmiA-AmiAH. The predicted sizes of AmiAH precursor (p) and mature (m) forms are 34 kDa and 28.9 kDa respectively. A form corresponding to AmiAH degradation product is indicated with an asterisk in the Δ*tatC* strain. The molecular masses (in kilodaltons) are indicated on the left of the gels. Full-length blots are presented in Supplementary Fig. 4 (**D**) The immunoblot bands from panel C were quantified from 6 experiments by densitometry analysis using the ImageQuant software.

Absence or insufficient protein synthesis of signal peptide-AmiA constructs could prevent complementation of the Δ*ssamiAC* mutant, and be interpreted as an absence of transport. Therefore, to monitor the production of protein fusions constructed later in this work we modified the original ssAmiA-AmiA fusion with the addition of a C-terminal hexa-histidine (His_6_) epitope-tag (ssAmiA-AmiAH). We showed that the new ssAmiA-AmiAH fusion is still functional because it allows the restoration of a wild type phenotype (SDS resistance and single cell formation) when produced in Δ*ssamiAC* (Fig. 3A and B). Importantly ssAmiA-AmiAH did not complement the Δ*tatC* mutation (a *tat* null mutant) for SDS sensitivity and chain formation indicating that AmiAH activity is strictly Tat dependent (Fig. 3A). The ssAmiA-AmiAH protein fusion was identified by immunoblot analysis using anti-His_6_ antibodies in wild type (WT), Δ*tatC* and Δ*ssamiAC* strains (Fig. 3C and D). Whereas the WT and Δ*ssamiAC* strains showed the precursor and the mature forms of ssAmiA-AmiAH in similar amounts (annotated p and m), the Δ*tatC* cells showed mostly the precursor form indicating a role for the Tat pathway in AmiAH export to the periplasm. This interpretation was confirmed after fractionation of WT cells since the precursor form remained associated with spheroplasts while the mature form was released into the periplasmic fraction (Fig. S1 lanes 3 and 4). In contrast in Δ*tatC* cells both forms remained associated with the spheroplast fraction indicating that the faster migrating band (indicated with an asterisk) is not an exported form of AmiAH but rather a cytoplasmic degradation product that migrates to the same position as the mature AmiAH (Fig. S1 lanes 5 and 6). This was confirmed by Proteinase K accessibility experiment: both protein bands were intact after Proteinase K treatment of spheroplasts but were degraded once cells were lyzed with Triton X-100 (Fig. S1 lanes 8 and 10). This indicates that both forms are located in the cytoplasm. Altogether, these results indicate that AmiA carrying a C-terminal histidine tag, AmiAH, is exported by the Tat system and that its activity can be used as an efficient reporter of Tat translocation.

### Experimental validation of the amidase Tat reporter assay for *P. aeruginosa* signal peptides

**Figure 4:**
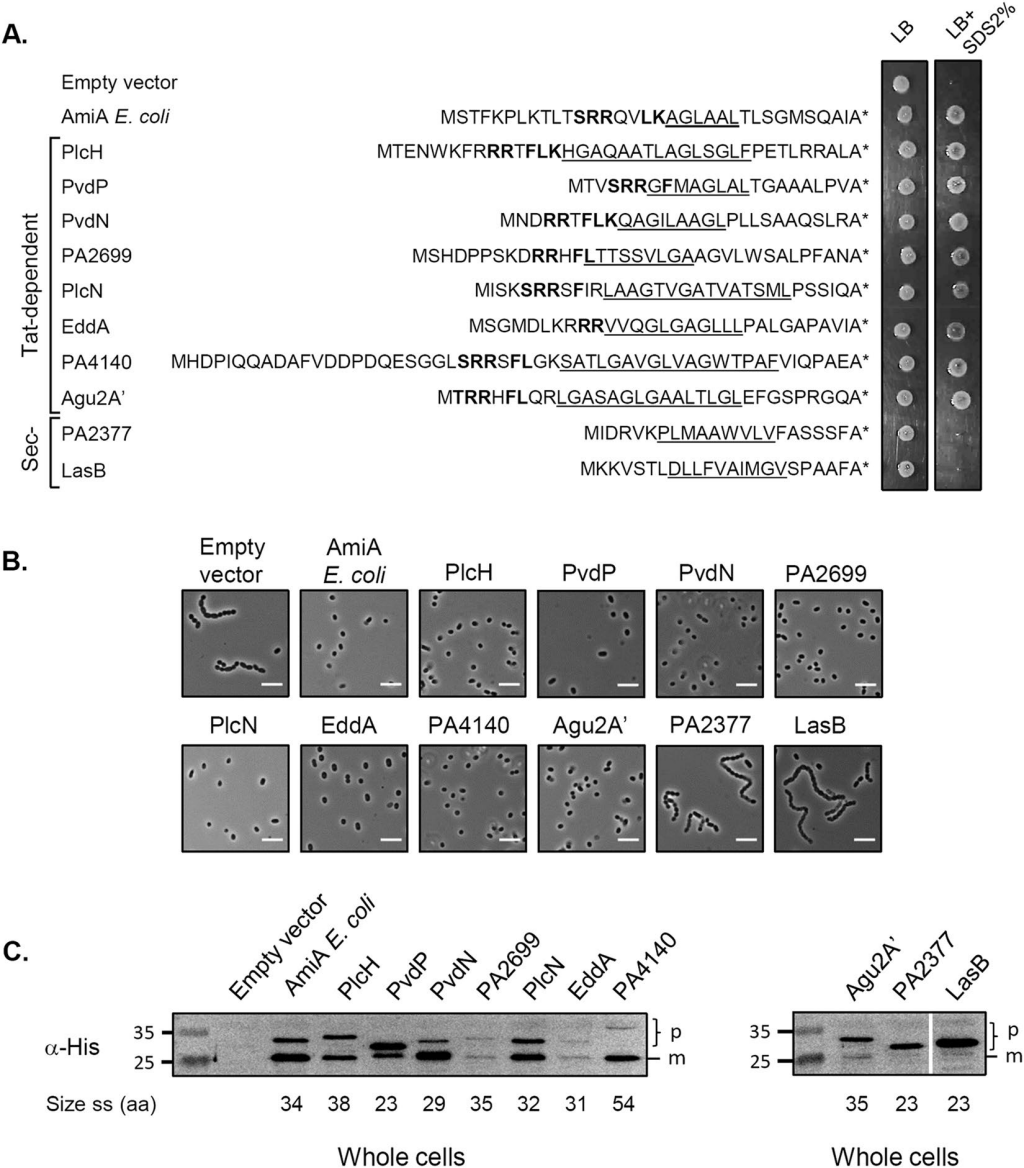
The amidase assay discriminates between *P. aeruginosa* Tat- and Sec-dependent signal peptides. SDS viability assay **(A**), chain formation (**B**), and immunoblot analysis (**C**) of *E. coli* Δ*ssamiAC* mutant (MCDSSAC) strain carrying pUNI-PROM (Empty vector), pssAmiA-AmiAH (producing *E. coli* AmiA with an His_6_ epitope-tag) or derivatives of pssAmiA-AmiAH where AmiA signal sequence has been replaced by the putative signal sequences of PA14 *P. aeruginosa* Tat-dependent and Sec-dependent known substrates. For each putative signal sequence the most likely twin-arginine motif is indicated in bold, the hydrophobic region is underlined and the predicted signal peptidase cleavage site is indicated by a star. For SDS-resistance assay, strains were grown aerobically and 5 μl were spotted on LB plate containing 2% (w/v) SDS where indicated. For microscopic observations the white bar represents 10 μm. For immunoblot analysis whole cell extracts were separated on a 12% SDS-PAGE gel and blots were probed with anti-his tag (His_6_). The predicted sizes of each signal peptide are indicated in amino acid at the bottom of the gel and the unprocessed (p) and processed (m) forms are indicated on the right of the gel. The molecular weight standards (in kilodaltons) are indicated on the left of the gels. The white line separates lanes from non-adjacent part of the same gel. Full-length blots are presented in Supplementary Fig. 4.

**Figure 5:**
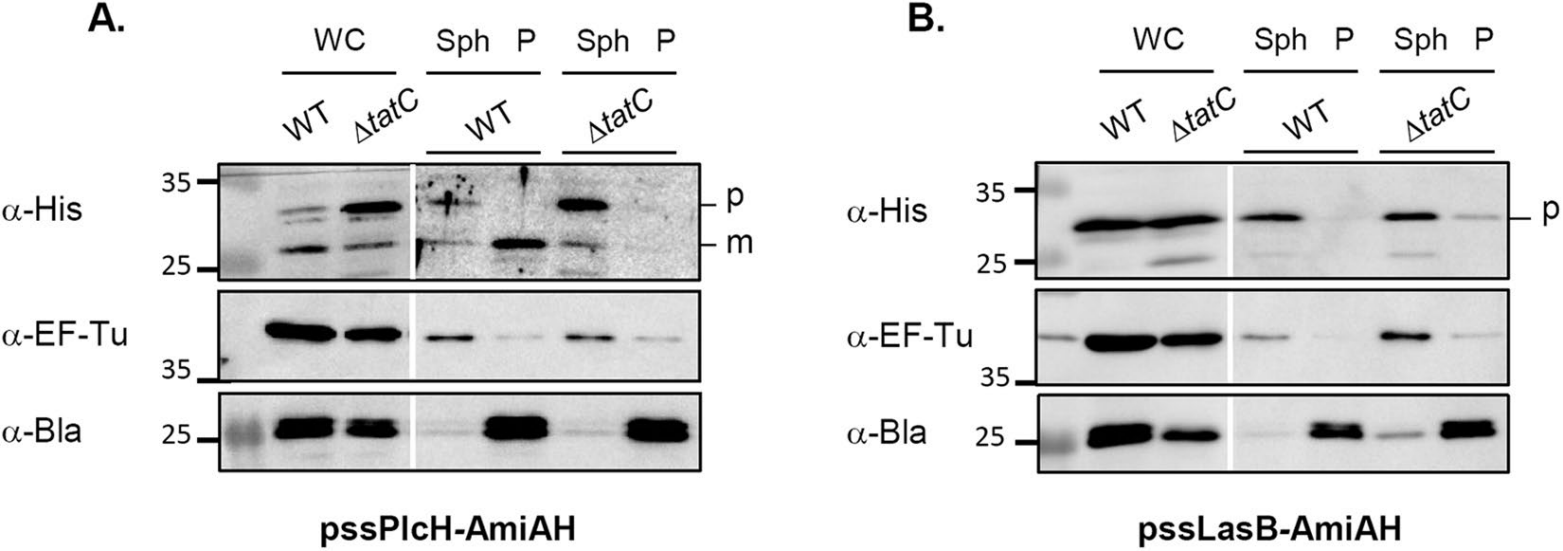
Localization of AmiAH fused to the archetypical Tat and Sec-dependent signal peptides. Immunoblot analysis of WT (MC4100) and Δ*tatC* (B1LK0) strains carrying pssPlcH-AmiAH (**A**) or pssLasB-AmiAH (**B**) after cell fractionation (where *E. coli* AmiA signal peptide has been replaced with *P. aeruginosa* PlcH or LasB signal peptide and where AmiA carries an His_6_ epitope-tag). Whole cell extracts (WC), spheroplasts (Sph) and periplasm (P) were separated on a 12% SDS-PAGE gel. Blots were probed with anti-his tag (His_6_), anti-elongation factor thermo unstable (EF-Tu) and anti-beta lactamase (Bla) antibodies. EF-Tu (43.3 kDa) is used as a cytoplasmic control and Bla (28.9 kDa) as a periplasmic control. The predicted sizes of ssPlcH-AmiAH unprocessed (precursor p) and processed (mature m) forms are 32.9 kDa and 28.6 kDa respectively while the predicted size of ssLasB-AmiAH unprocessed (precursor p) is 31.3 kDa and are indicated on the right of the gel. The molecular masses (in kilodaltons) are indicated on the left of the gels. A white line separates lanes from non-adjacent part of the same gel and with different exposures. Full-length blots are presented in Supplementary Fig. 4.

Next we tested whether the amidase reporter system can recognize *P. aeruginosa* Tat signal peptides and robustly differentiate between Sec and Tat signal peptides. Fusions to all known *P. aeruginosa* Tat substrate signal peptides were constructed and tested for rescue of the Δ*ssamiAC* mutant SDS sensitivity and chain formation phenotypes (Fig. 4A and B). All constructs were made by fusion of each signal peptide to the AmiAH mature domain (without signal peptide) thus creating protein fusions bearing the native *E. coli tatABC* promoter and Shine-Dalgarno (SD) sequence with identical distance between the SD sequence and initiation codon^23^. The production of each protein fusion was monitored by immunoblot analysis of whole cell fractions (Fig. 4C). Notably we found that Tat signal peptides from all known *P. aeruginosa* Tat substrates allowed the export of the AmiAH reporter protein and rescued the Δ*ssamiAC* mutant phenotypes, even in the case of the least produced ones (PA2699 and EddA). To assess the reporter assay specificity we next tested its ability to discriminate between *P. aeruginosa* Sec and Tat signal peptides by constructing protein fusions between AmiAH and two Sec signal peptides (LasB and PA2377). We showed that neither of these Sec signal peptides rescued chain formation nor growth of the reporter strain on SDS (Fig. 4A and B) even if they were produced correctly (Fig. 4C). Finally, we studied the cellular localisation of AmiAH fused to the archetypal Tat (PlcH) and Sec (LasB) signal peptides after fractionation of a WT and a Δ*tatC* mutant (Fig. 5). Whereas AmiAH fused to PlcH Tat signal peptide was recovered in the periplasm as a mature form in WT cells, it accumulated clearly as a precursor in the spheroplasts of a Δ*tatC* mutant. This indicates that when fused to a Tat signal peptide AmiAH was exported to the periplasm in a strictly Tat-dependent manner (Fig. 5A). When AmiAH was fused to the Sec signal peptide of LasB, a single band was observed at the expected size of the precursor in WT and Δ*tatC* strains (Fig. 5B). After cell fractionation, this band stayed associated with the spheroplasts. This indicates that AmiAH was not exported to the periplasm when fused to a Sec signal peptide but was instead retained in the spheroplasts. We conclude from this set of experiments that *P. aeruginosa* Tat signal sequences can be efficiently and specifically recognized by the *E. coli* Tat machinery and that the *E. coli* amidase reporter assay can distinguish between the Sec and Tat-dependent nature of *P. aeruginosa* N-terminal regions.

### Systematic analysis of the *P. aeruginosa* candidate signal peptides by the amidase reporter assay

**Figure 6:**
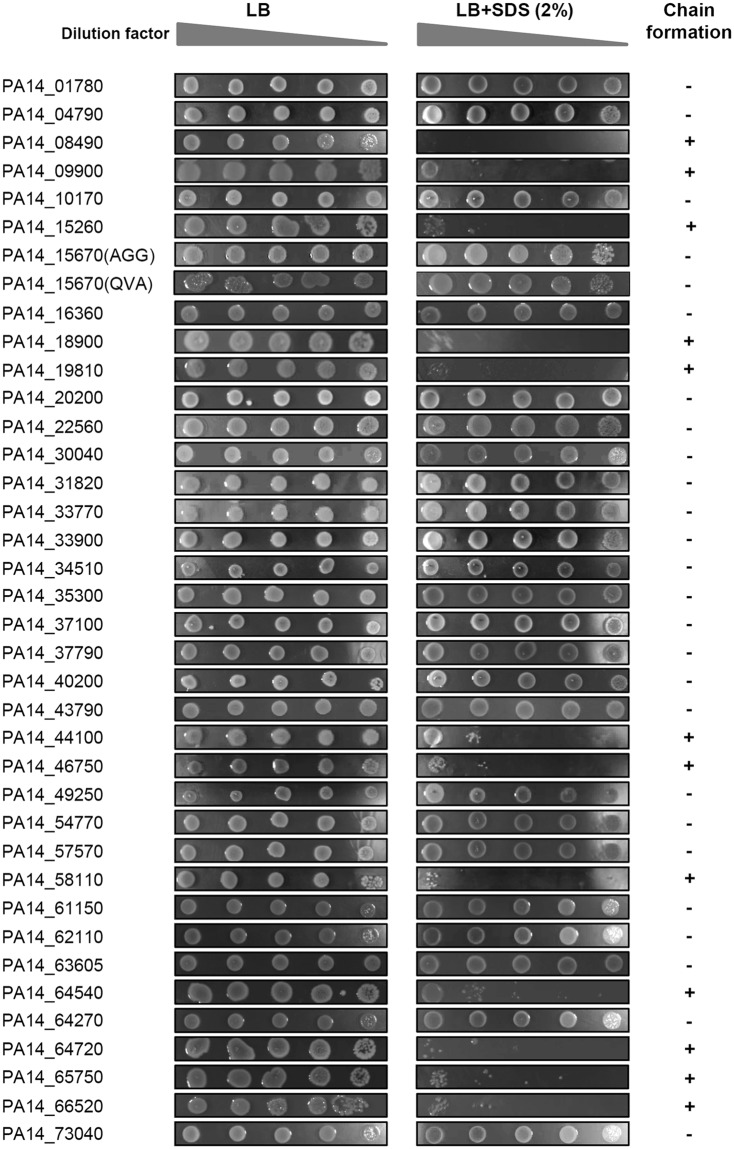
Screening of the putative Tat candidates by the amidase reporter assay. SDS viability assay of *E. coli* Δ*ssamiAC* mutant (MCDSSAC) strain carrying derivatives of pssAmiA-AmiAH where AmiA signal sequence has been replaced by the 39 candidate Tat signal peptides identified *in silico*.

To determine the Tat-dependence of the 37 newly identified *P. aeruginosa* candidate Tat signal peptides we fused each candidate to the AmiAH mature domain and tested their capacity to engage the Tat pathway with the amidase reporter assay. The length of each candidate signal peptide was defined after prediction of signal peptidase cleavage site as described in Material and methods. For one of the candidates, PA14_15670, two versions of the region encoding the putative Tat signal peptide were cloned because two possible signal peptidase cleavage sites were predicted (AGG* and QVA*) (Table 1A). We systematically analyzed the SDS sensitivity and chain formation phenotypes of Δ*ssamiAC* carrying each of the 38 fusion proteins (Fig. 6). Out of the 38 constructs tested, 26 clearly complemented the Δ*ssamiAC* mutation indicating that they were real Tat signal peptides. Notably both versions of the PA14_15670 signal peptide (constructed with two different cleavage sites) restored the Δ*ssamiAC* mutant phenotypes. Observation of the production and export of AmiAH fused to these two signal peptides indicate however that the export is slightly more efficient when AmiAH is fused after the AGG codon (Fig. S2). In contrast, 12 out of the 37 Tat candidates tested did not complement the Δ*ssamiAC* mutation even if they were correctly produced (Figs 6 and S3). This indicates that these candidates are not authentic Tat signal peptides. Notably, these include four candidates that contain the unusual KR (PA14_44100 and PA14_09900) and RK (PA14_46750 and PA14_64720) motifs. In all, this approach led to the identification of 25 new Tat signal peptides in *P. aeruginosa* bringing the total number of *P. aeruginosa* Tat substrates to 33.

### Identification of a new periplasmic Tat substrate with a missassigned start codon

Recently, a comparative proteomic approach allowed us to identify a new Tat substrate in *P. aeruginosa* with a missassigned start codon, PA2699^29^. Our *in silico* approach misses such substrates because the screening was performed on the *P. aeruginosa* predicted proteome. Consequently, to identify other putative Tat substrates that may have missassigned start codons we ran a modified TATFIND prediction program on all PA14 open reading frames (instead of the *P. aeruginosa* protein database) bigger than 150 base pairs starting with an ATG and having two codons encoding RR, RK and KR within the first 120 nucleotides. This analysis led to the identification of 523 potential Tat peptides encoded by the 33416 putative ORFs tested (Table S1). As expected, amongst these we found all the putative Tat signal peptides starting with an ATG that were identified with the previous analysis. Next we manually curated the remaining hits to identify putative Tat signal peptides by removing (i) sequences in the opposite orientation to an annotated gene, (ii) sequences not directly in frame with an annotated gene and (iii) sequences further than 150 nucleotides from the start codon of an annotated gene. The remaining sequences were then analyzed for the presence of signal peptides using the SignalP, TatP and PRED-TAT algorithms and scrutinized for the presence of a putative SD sequence upstream of the putative missassigned start codon. This analysis led to the identification of one more Tat candidate, *PA14_70330* or SphC. Of note PA2699, which we identified previously with a missassigned initiation codon, was not identified in this analysis because the TATFIND program does not recognize the PA2699 Tat signal peptide^29^.

**Figure 7:**
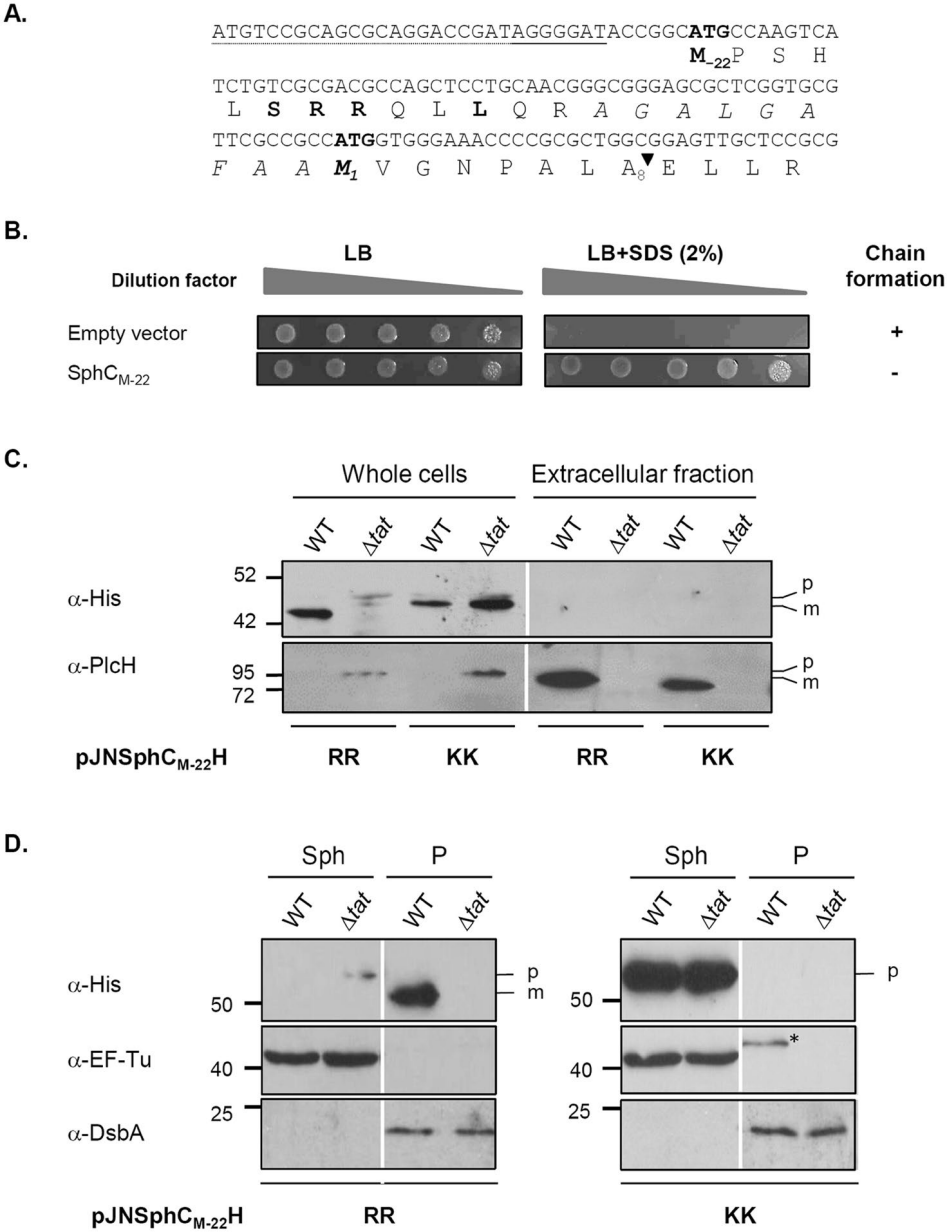
PA14_70330 is a new periplasmic Tat substrate with a missassigned start codon. (**A**) Nucleotide sequence of the *PA14_70330/sphC* upstream region containing the start codon annotated on the PA14 genome (M_1_) and the putative missassigned start codon (M_-22_) identified in this study. Deduced amino acid sequence of SphC is shown below the nucleotide sequence. A putative SD sequence based on the canonical AGGAGGU is underlined. The forward primer used to PCR amplifies *sphC* and its promoter region is underlined (dotted line). On the putative SphC protein sequence, the Tat motif is indicated in bold, the hydrophobic region is in italics, and the signal peptidase I cleavage site is indicated by an arrow. (**B**) SDS viability assay of *E. coli* Δ*ssamiAC* mutant (MCDSSAC) strain carrying pUNI-PROM (empty vector) and pssSphC_M-22_-AmiAH (SphC_M-22_). (**C**) and (**D**) Immunoblot analysis of whole cells, extracellular fraction, spheroplasts (Sph) and periplasmic (P) fractions of PA14 (WT) and PA14Δtat (Δ*tat*) carrying pJNSphC_M-22_H (RR) and pJNSphC_M-22_H-KK (KK), grown in phosphate depleted medium (**C**) or in rich medium (**D**) supplemented with arabinose as described in material and methods. The predicted sizes of SphC_M-22_H precursors (p) and mature (m) forms are respectively 52.6 kDa and 49.5 kDa. PlcH (precursor: 82.7 kDa, mature form: 78.3 kDa) is used as supernatant quality control. The protein disulfide isomerase DsbA (23.3 kDa) and EF-Tu (43.3 kDa) are shown as periplasmic and cytoplasmic controls respectively. The asterisk in panel D indicates an aspecific band. Molecular weight markers are indicated on the left of the blot. A white line separates lanes from different gels (**C**) or from non-adjacent part of the same gel (**D**). Full-length blots are presented in Supplementary Fig. 4.

Analysis of the nucleotide sequence of the annotated *sphC* gene showed the presence of another ATG codon 66 nucleotides upstream, with a typical SD sequence, and in frame with the annotated start codon of *sphC* (Fig. 7A). The utilization of an algorithm developed to identify bacterial start codons showed that the upstream ATG is better than the annotated ATG with a score of 28 compared with a score of 17 (a score of ≥26 is considered an initiation codon)^42^. Moreover, if the upstream ATG is used as a translation start (M_−22_) SphC is then predicted to possess a Tat signal peptide by the three prediction programs, with a clear Tat motif SRRQLL, a hydrophobic stretch and a typical leader peptidase cleavage site ALA (Fig. 7A).

First, to test the capacity of SphC putative N-terminal region (from M_−22_ to A_8_) to act as a Tat signal peptide, we fused it to AmiAH mature domain and introduced the construct into the Δ*ssamiAC* reporter strain. Production of the SphC_M-22_ signal peptide-AmiAH fusion confered SDS resistance and rescued the chain formation phenotype of the reporter strain indicating that SphC putative N-terminal region contains an active Tat signal peptide (Fig. 7B). Next, we decided to confirm the dependence of SphC on the Tat system *in vivo* in *P. aeruginosa*. We constructed a C-terminal His_6_ epitope-tagged version of the protein (SphC_M-22_-H) and assessed its subcellular localization in the WT and Δ*tat*C strains. Immunobloting identified SphC_M-22_-H in both strains in whole cells rather than in the extracellular fraction indicating that the protein is not secreted (Fig. 7C). As expected for a Tat-dependent substrate, SphC_M-22_H was present as a slower migrating band (precursor) in the Δ*tat* mutant in comparison to the WT strain where SphC_M-22_H migrated at the expected size for the mature form. In contrast when we replaced the two essential arginines of the Tat consensus sequence by two lysines (thus blocking Tat export) SphC_M-22_H-KK appeared as a precursor form in both WT and Δ*tat* mutant strains confirming the Tat dependent export of SphC_M-22_H *in P. aeruginosa* (Fig. 7C). Finally, upon cellular fractionation, SphC was found in the periplasmic fraction of the WT strain and accumulated as a precursor form in a *tatC* background or when the twin-arginines where replaced by lysines (Fig. 7D). Altogether, our *in silico* approach and *in vivo* results reveal the existence of another Tat substrate with a missassigned start codon, SphC, and demonstrate that it is a periplasmic protein rather than a cytoplasmic protein. In conclusion, our previous data and the work presented here show that *P. aeruginosa* possesses 34 Tat substrates of which 2 have missassigned start codons.

## Discussion

In this work we employed a combination of a stringent *in silico* analysis and *in vivo* reporter fusion assays to reveal that *P. aeruginosa* encodes 34 proteins with functional Tat signal peptides and by extrapolation strongly suggesting that *P. aeruginosa* contains 34 Tat substrates (Table 2). Of these 34 proteins, 8 were previously identified as Tat substrates, and the signal peptides of the remaining 26 were experimentally shown to allow Tat export for the first time in this study. Previously, manual screens of the *P. aeruginosa* genome identified 18 and 27 putative Tat signal peptides, with none or little experimental validation^33,38^. Here, we used a stringent *in silico* approach to identify potential Tat substrates: selecting only those substrates identified by at least two independent algorithms, relaxing the search criteria to allow identification of unusual Tat signal peptides, and additionally scanning the translated genome to identify substrates with missassigned start codons. Interestingly, compared to previous studies on *P. aeruginosa*, we found 15 new substrates, but we also did not identify all the predicted substrates previously reported. This is not surprising, considering that these studies were performed with a large manual component, and that even the expert algorithms that we used did not fully overlap in their predictions (Fig. 1). Therefore, the 34 experimentally validated Tat substrates that we identify represent the high quality core complement of *P. aeruginosa* Tat substrates. Our findings do not exclude that the *P. aeruginosa* proteome may harbor a limited number of other atypical Tat substrates. Interestingly, 24 of the Tat signal peptides we validated were predicted by the three Tat prediction programs, while four were predicted by TATFIND and TatP, one by TATFIND and PRED-TAT and three by TatP and PRED-TAT (Fig. 1). The signal peptides of SphC and PA2699 whose start codons are missassigned (Fig. 7 and ref.^29^) were predicted as Tat signal peptide respectively by the three prediction programs and by TatP and PRED-TAT. Altogether this shows the limitation of single prediction programs and highlights the need for experimental validation of Tat export.

**Table 2:** 34 Tat-dependent proteins in *P. aeruginosa*.

**PA14 gene id**	**PAO1 gene id**	N-terminal signal sequence^a^	Predicted gene product^b^	Predicted metal clusters/cofactors^b^	Functional identification^b^	Gene ontology - Biological process^b^	Taille (kDa)^b^	Tat-dependance
**Secreted factors**								
PA14_10370	PA4140	MHDPIQQADAFVDDPDQESGGL**SRR**S**FL**GKSATLGAVGLVAGWTPAFVIQPAEA*	Cholesterol oxidase	Flavin nucleotides	Fatty acid and phospholipid metabolism	Oxidation-reduction process	65.3	Ball *et al*.^29^
PA14_13330	PA3910	MSGMDLK**RR**RVVQGLGAGLLLPALGAPAVIA*	Phosphodiesterase /alkaline phosphatase, EddA	None predicted	Secreted Factors (toxins, enzymes, alginate)	Unknown	58.7	Ball *et al*.^29^
PA14_21110	PA3319	MISK**SRR**S**F**IRLAAGTVGATVATSMLPSSIQA*	Non-hemolytic phospholipase C, PlcN	None predicted	Secreted Factors (toxins, enzymes, alginate)	Lipid catabolic process	77.2	Voulhoux *et a*l.^32^
PA14_29230^c^	PA2699	MSHDPPSKD**RR**H**FL**TTSSVLGAAGVLWSALPFANA*	Hydrolase	None predicted	Putative enzymes	Unknown	68	Ball *et al*.^29^
PA14_53360	PA0844	MTENWKFR**RR**T**FLK**HGAQAATLAGLSGLFPETLRRALA*	Hemolytic phospholipase C, PlcH	None predicted	Secreted Factors (toxins, enzymes, alginate)	Pathogenesis	82.7	Voulhoux *et al*.^32^
**Iron acquisition**								
PA14_10170	PA4159	MP**TRR**RSALPLLALALSLFATLAAA*	Iron-enterobactin transporter periplasmic binding protein, FepB	None predicted	Transport of small molecules	Ferric-enterobactin transport	32.1	This work
PA14_33720	PA2394	MND**RR**T**FLK**QAGILAAGLPLLSAAQSLRA*	Aminotransferase, PvdN	None predicted	Adaptation, Protection	Pyoverdine biosynthetic process	48	Voulhoux *et al*.^34^
PA14_33740	PA2392	MTV**SRR**G**F**MAGLALTGAAALPVA*	Tyrosinase, PvdP	Di-copper centre	Adaptation, Protection	Pyoverdine biosynthetic process	62.3	Nadal-Jimenez *et al*.^35^
PA14_33770	PA2389	MRRTRS**TRR**AL**L**VAVCLSPLIALA*	Hypothetical protein, PvdR	None predicted	Transport of small molecules	Pyoverdine biosynthetic process	42.1	This work
**Energy Metabolism**								
PA14_20200	PA3392	MSDDTKSPHEETHGLN**RR**G**F**LGASALTGAAALVGASALGSAVVGREARA*	Nitrous-oxide reductase, NosZ	Copper centre	Energy metabolism	Denitrification pathway	70.6	This work
PA14_49250	PA1174	MNL**TRR**E**F**A**K**ANAAAIAAAAAGLPILVRA*	Nitrate reductase catalytic subunit, NapA	Molybdopterin [4Fe-4S]	Energy metabolism	Nitrate assimilation	93.5	This work
PA14_57570	PA4431	MSNDGVNAG**RR**R**FL**VAATSVVGAAGAVGAAVPFVGSWFPSAKAKA*	Cytochrome c reductase, iron-sulfur subunit	[2Fe-2S]	Putative enzymes	Oxidation-reduction process	20.8	This work
PA14_63605	PA4812	MDMN**RR**Q**F**F**K**VCGIGLGGSSLAALGMAPTEAFA*	Formate dehydrogenase-O, major subunit, FdnG	Molybdopterin [4Fe-4S]	Energy metabolism	Anaerobic respiration	114	This work
**Other activities**								
PA14_01780	PA0144	MSRSNGSSS**RR**T**FL**RLAALLLPAGALLGSLPGVRA*	Nucleoside 2-deoxyribosyltransferase	None predicted	Deoxyribonucleoside monophosphate catabolic process	Hypothetical, unclassified, unknown	22.3	This work
PA14_15670	PA3768	MTF**TRR**QV**L**GGLAGLAVVGLGAGG*	Metallo-oxidoreductase	Copper centre	Putative enzymes	Oxidation-reduction process	51.5	This work
PA14_31820	PA2531	MPAL**SRR**S**F**VTLTALASSSILLSPRAFA*	Aminotransferase	None predicted	Amino acid biosynthesis and metabolism	Biosynthetic process	40.7	This work
PA14_33900	PA2378	MKRSFPDDLVIGNL**SRR**G**FLK**GVGATGVLLVAANWGWRDALA*	Aldehyde dehydrogenase	Molybdopterin	Putative enzymes	Oxidation-reduction process	83.7	This work
PA14_37100	PA2124	MHQPENPA**RR**TL**L**AQTVAGSAALALGSLLGGAPGVASA*	Dehydrogenase	Flavin nucleotides NAD^+^	Putative enzymes	Oxidation-reduction process	57.3	This work
PA14_37790	PA2065	MHRT**SRR**T**F**V**K**GLAATGLLGGLGLWRAPAWA*	Copper resistance protein A, CopA	Copper centre	Adaptation, Protection	Oxidation-reduction process	67.1	This work
PA14_40200	PA1880	MNSKIDLSNALPG**SRR**G**FLK**GAAVVGLTIGFQWSGARRALA*	Oxidoreductase	Molybdopterin	Putative enzymes	Oxidation-reduction process	77.6	This work
PA14_43790	PA1601	MSLANP**SRR**G**FLK**AGGLLLVTVNLPAPLLALA*	Aldehyde dehydrogenase	Molybdopterin	Putative enzymes	Oxidation-reduction process	81.4	This work
PA14_48450	Not_conserved	M**TRR**H**FL**QRLGASAGLGAALTLGLEFGSPRGQA*	Peptidyl-arginine deiminase, Agu2A’	None predicted	Putative enzymes	Putrescine biosynthetic process	40.5	Williams *et a*l.^36^
PA14_61150	PA4621	MSNRDI**SRR**A**FL**QGGLIAGVGVTLAPLGSQAFA*	Oxidoreductase	Molybdopterin	Putative enzymes	Oxidation-reduction process	103.7	This work
PA14_62110	PA4692	MLIKIPSRSDCSESEVTSETLYL**SRR**RL**L**GASFAGLALASGLPRLGFADE*	Sulfite oxidase subunit, YedY	Molybdopterin	Hypothetical, unclassified, unknown	Oxidation-reduction process/Protein repair	38.2	This work
PA14_70330	PA5327	MPSHL**SRR**QL**L**QRAGALGAFAAMVGNPALA*	Oxidoreductase, SphC	Flavin nucleotides	Energy metabolism	Oxidation-reduction process	49.4	This work
PA14_73040^d^	PA5538	MK**RR**RL**L**QSLLAGLALQPFLAASA*	N-acetylmuramoyl-L-alanine amidase, AmiC	Zn^2+^	Cell wall**/**LPS**/**capsule	Peptidoglycan catabolic process	42.9	This work
**Hypothetical**								
PA14_04790	PA0365	MSDTTLESAGL**SRR**SLM**K**VGLIGGAFLATA*	Hypothetical protein	None predicted	Hypothetical, unclassified, unknown Membrane proteins,	Unknown	19.6	This work
PA14_16360	PA3713	MTI**SRR**D**FL**NGVALTIAAGLTPAEILRA*	Hypothetical protein	Flavin nucleotides NAD^+^	Putative enzymes	Oxidation-reduction process	68.9	This work
PA14_22560	PA3222	MN**RR**SA**L**AALHGGALLFGLTGVFGKLASA*	Permease	None predicted	Membrane proteins	Unknown	31	This work
PA14_30040	PA2635	MSLEKKDAILFGDGDELPSNHSNNPHMNDLIAGLG**RR**QV**L**AGGAALGALAFLGVALPASA*	Hypothetical protein	None predicted	Hypothetical, unclassified, unknown	Unknown	73.8	This work
PA14_34510	PA2328	MCLDDPTH**SRR**DI**LK**LAALLSAAGALPLLSSLQARA*	Hypothetical protein	None predicted	Transport of small molecules	Unknown	43.7	This work
PA14_35300	PA2264	MPDDKAVNG**RR**D**FL**RKTLTVIPAVTLAGYGVGHAMQTPA*	Hypothetical protein	None predicted	Hypothetical, unclassified, unknown	Unknown	26.4	This work
PA14_54770	PA0735	MNRN**RR**NALIAGSLLLLAANLAALGGVAWNRS*	Hypothetical protein	None predicted	Hypothetical, unclassified, unknown	Unknown	30.8	This work
PA14_64270	PA4858	MK**RR**SL**LK**AFTLSASIASMGLSWSIQA*	Hypothetical protein	None predicted	Transport of small molecules	Amino acid transport	46.2	This work

^a^Amino acids matching those of the twin arginine motif are indicated in bold, the hydrophobic domain is underlined and the putative cleavage site is indicated by and asterisk

^b^Predictions found on the pseudomonas.com website or inferred by homology and sequence analysis.

^c^Manually reassigned start codon.

^d^PA14_73040 is annotated on the *Pseudomonas* genome as AmiA but is closer to AmiC^66^. Protein sequence analysis indicate that PA14_73040 contains all the amino acids essential to a potential Zn^2+^ binding site always present in amidases.

Here we used an improved amidase reporter assay that we initially developed in *E. coli*^23^. We use it for the first time for the systematic screening of substrates identified *in silico*. We improved the assay with the addition of a C-terminal epitope tag to AmiA thus allowing detection and localization of the various protein fusions constructed. Importantly, the epitope tag allowed us to show that AmiA export is strictly Tat-dependent when fused to a Tat signal peptide and that the assay works even when the protein fusion is not strongly produced highlighting the sensitivity of the assay (Fig. 3). In addition we also showed that the amidase assay is robust, and can reliably discriminate Sec from Tat signal peptides (Fig. 4). Notably, while the assay was developed for *E. coli* signal peptides, we show here that it reliably recognizes *P. aeruginosa* signal peptides (Fig. 4). This is undoubtedly due to the fact that the substrates recognition sites on the *E. coli* and *P. aeruginosa* TatC protein (the receptor of the system) are perfectly conserved^43^. We therefore anticipate that the *E. coli* amidase reporter assay will be broadly applicable for the identification of Tat signal peptides from other organisms.

**Figure 8:**
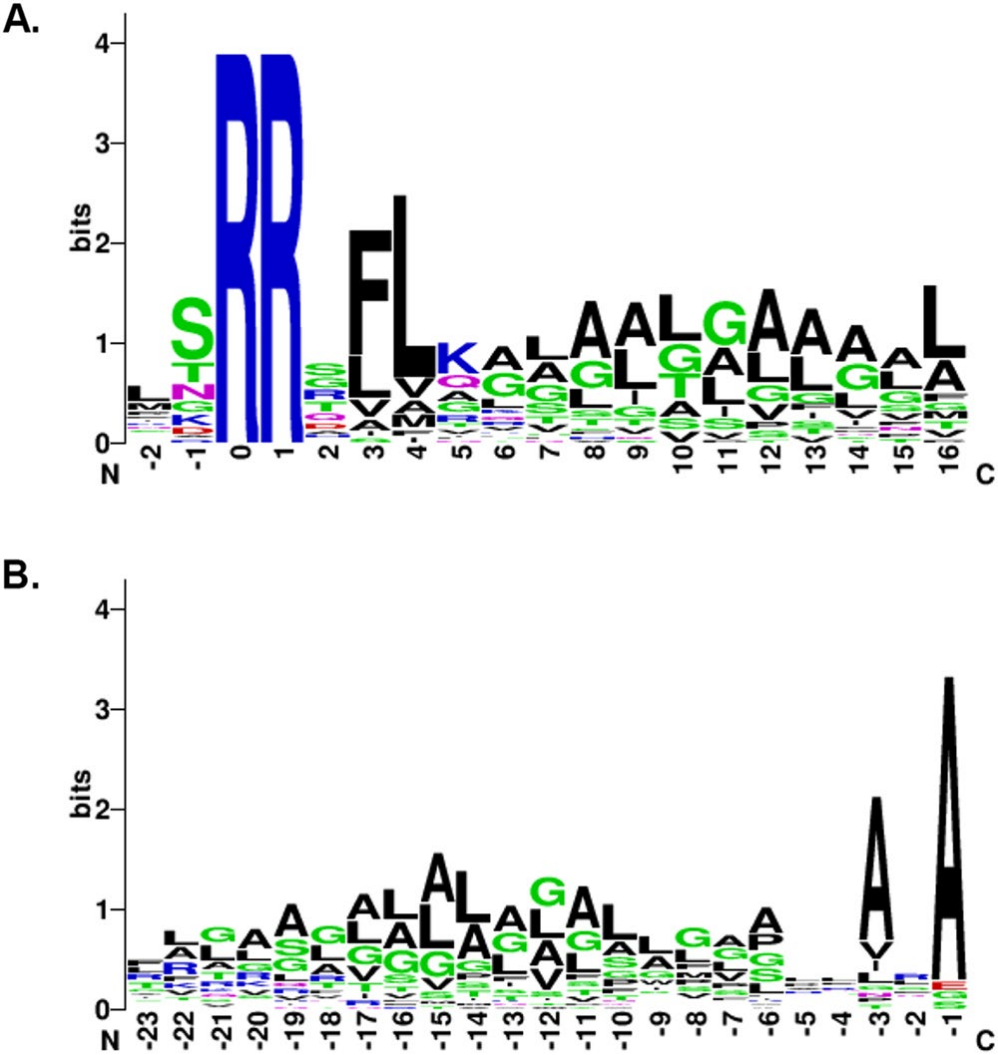
*P. aeruginosa* Tat signal peptide logo derived from signal peptides identified in this work and found in Table 2. Sequence logos of signal peptides aligned either (**A**) from two amino acids before the conserved twin-arginine or **(B**) from their cleavage sites. Amino acids are colored according to their chemical properties: polar amino acids (G, S, T, Y, C, Q, N) are green, basic (K, R, H) blue, acidic (D, E) red and hydrophobic (A, V, L, I, P, W, F, M) amino acids are black. The overall height of the stack indicates the sequence conservation at that position, while the height of symbols within the stack indicates the relative frequency of each amino at that position.

To visualize the sequence information content in *P. aeruginosa* Tat signal peptides we generated sequence logos with the 34 Tat signal peptides, from two amino acids before the twin-arginine dipeptide and from the signal peptidase I cleavage site (Table 2 and Fig. 8). Notably, the hallmark of Tat signal peptide can be visualized with the presence of the S/TRRXFLK motif where the twin-arginine dipeptide is strictly conserved. The FLK amino acids are also highly conserved despite the lysine not being present at a frequency in excess of 50% as defined previously^7^ but rather at a frequency around 32% (Fig. 8A). In addition the logo shows that the H-region, starting after the Tat motif (Fig. 8B residues −6 to −22), contains a significant number of glycine residues as is usually the case in Tat signal peptides. It also contains the typical leucine and alanine residues present in the hydrophobic region. Tat signal peptides are distinctly longer than Sec signal peptides with an average length of 38 amino acids compared to 24 amino acids^44^. Here the signal peptides vary between 23 and 60 amino acids with an average length of 34 amino acids. As previously noted^8^, the extended length arises mainly from a longer N-region (Table 2). Earlier studies suggested that Tat signal peptides contained positively charged amino acids in the C-region that could play the role of a “Sec avoidance signal”^10^. Notably, the sequence logo does not illustrate a high occurrence of lysine or arginine corresponding to such motif and only few *P. aeruginosa* Tat signal peptides contain positively charged amino acids in the C-region (Fig. 8B and Table 2). Finally, the sequence alignment shows that the canonical signal peptidase I cleavage site AXA is largely present in the C-region. Interestingly, we did not find any signal peptides with a signal peptidase II cleavage site as can be found in Tat signal peptides from *Streptomyces* and haloarchae^9,22^. Altogether we conclude from this analysis that *P. aeruginosa* Tat signal peptides share most of the characteristics of classical Tat signal peptides.

The 34 Tat substrates identified previously and in this work are listed Table 2 along with their predicted function, the biological process they are involved in when known, their size and their predicted association with a metal cluster or a cofactor. For a long time the Tat system was thought to be mainly involved in the transport of proteins containing cofactors that needed to be folded in the cytoplasm before export, because the biosynthetic machinery for these cofactors was cytoplasmic. However, later studies showed that the Tat system was also involved in the export of cofactor-less proteins which for unknown reasons need to be folded before their export. Here half (17 out of 34) of the *P. aeruginosa* Tat substrates are predicted to contain cofactors. These cofactors can be classified into nucleotide based cofactors such as molybdopterin (7 substrates) and flavin (4 substrates), and metal clusters such as iron-sulfur clusters (3 substrates), copper centers (4 substrates) and zinc clusters (1 substrate). For comparison, in *E. coli* K12 where Tat substrates are the most characterized, 68% of Tat substrates (19 out of 28) bind cofactors of similar nature^44^. The Tat pathway is notorious for its versatility. Indeed, in addition to the export of folded soluble proteins, it can also export lipoprotein^9,22^, protein complexes where only one of the partners has a Tat signal peptide^45^, and proteins with N- or C-terminal anchors^46,47^. None of the *P. aeruginosa* Tat substrates are lipoproteins. One Tat substrate, FdnG, the major subunit of the formate dehydrogenase-O, is known to be exported as a complex with its partner FdnH (which is signal peptide-less) in other organisms. Once exported FdnH is probably attached to the inner membrane through a C-terminal transmembrane anchor^48^. Another of the *P. aeruginosa* Tat substrates is the Rieske iron sulfur protein, which is a typical Tat substrate whose N-terminal Tat signal peptide is not cleaved and is used as a transmembrane anchor instead^46^. The *P. aeruginosa* Tat system, in addition to transporting soluble proteins, therefore also appears capable of exporting protein complexes and proteins with N or C-terminal anchors.

As mentioned before Tat substrates have been best characterized in *E. coli* K12, as much for their identity as for their function^44^. Comparison of the *P. aeruginosa* Tat substrates with *E. coli* Tat substrates shows that they share the same range of sizes ranging from around 20 kDa (20.3 kDa for YbdK in *E. coli* and 20.8 kDa for PA14_57570 in *P. aeruginosa*) to around 110 kDa (FdnG for both organisms). Surprisingly only five Tat substrates are common between *P. aeruginosa* and *E. coli*: AmiC, CueO/CopA, FdnG, NapA and YedY. In addition to these *E. coli* orthologues, *P. aeruginosa* shares Tat substrates with known Tat substrates from other organisms. These include: the nitrous oxide reductase, NosZ, in *Ralstonia eutropha*^49^ and *Pseudomonas stutzeri*^50^, the Rieske protein in *Paracoccus denitrificans*^46^ and *Legionella pneumophila*^51^, the phosphodiesterase PhoD in *Bacillus subtilis*^52^, the phospholipases C PlcA and PlcB in *Mycobacterium tuberculosis*^21,53^. Analysis of the substrates with known function reveals that the *P. aeruginosa* Tat system is involved in a wide spectrum of functional classes centered on the adaptation of the bacteria to its niche and establishment of an infection. There are substrates involved in growth in the presence or absence of oxygen^54^, in survival during stress conditions (iron deficiency^55^, elevated levels of copper^56^…) and in pathogenesis^57,58^. Notably, a significant proportion of the Tat substrates we identified still have hypothetical or unknown functions. Future work will be devoted to understanding how these substrates promote *P. aeruginosa* survival and adaptation in a variety of ecological niches, including the eukaryotic host.

## Material and Methods

### *In silico* genome and sequence analysis

A modified version of TATFIND was used to screen the *P. aeruginosa* PA14 proteome for the presence of Tat signal peptides (File S1). For the identification of *P. aeruginosa* Tat substrates with TatP (http://www.cbs.dtu.dk/services/TatP) we specified the regular expression [RK][KR].[FGAVML][LITMVF] for output filtering. Identifiers with at least 1 “Yes” amongst the five scores represented in the output of TatP were selected as candidates Tat signal peptides.

For the identification of *P. aeruginosa* Tat substrates with PRED-TAT we ran the PRED-TAT (HMMER) program (available http://www.compgen.org/tools/PRED-TAT/supplement) on all PA14 predicted proteins then submitted the hits we obtained to the PRED-TAT webserver (http://www.compgen.org/tools/PRED-TAT/) using both the “original” and the “new” models. This analysis predicted 32 Tat substrates (Table S1).

For the identification of *P. aeruginosa* Tat substrates which might have a missassigned start codon, all PA14 ORFs of 50 amino acids starting with an ATG and having RR or RK or KR within the first 20 amino acids were extracted from a genbank file downloaded from https://www.ncbi.nlm.nih.gov/nuccore/NC_008463.1/ leading to 33416 sequences. The obtained amino acid sequences were then used as input to the modified TATFIND program.

For manual signal peptides prediction and analysis, SignalP3.0 (http://www.cbs.dtu.dk/services/SignalP-3.0/), TATFINDv1.4 (http://signalfind.org/TATFIND.html), TatP1.0 and PRED-TAT algorithms were used on the first 70 residues of *P. aeruginosa* PA14 proteins. Signal peptides domain prediction (N, H and C) was performed using SignalP 3.0 (HMM) and Phobius. Most likely signal peptide cleavage sites were predicted by SignalP3.0, TatP1.0 and PRED-TAT and after looking for sequence conservation of the cleavage site in orthologues if necessary. Identification of Shine-Dalgarno (SD) sequence was based on the AGGAGGT sequence. Identification of the most likely start codon was achieved using the Kolaskar and Reddy method that analyse the −18 to +18 nucleotides around the ATG/GTG^42^. Sequence logos were constructed using the web based application WebLogo and default settings http://weblogo.berkeley.edu/logo.cgi.

### Bacterial strains, plasmids and growth conditions

*E. coli* strains were cultured in Lysogeny broth (LB) with antibiotics as required (50 μg ml^−1^ ampicillin (Ap), 25 μg ml^−1^ kanamycin (Kan), 15 μg ml^−1^ gentamicin (Gm). The *E. coli* DH5α strain was used to propagate plasmids. Recombinant plasmids were introduced in the reference strain *P. aeruginosa* PA14 using pRK2013^59^ and transconjugants selected on *Pseudomonas* isolation agar (PIA, Difco Laboratories) supplemented with antibiotics as required (150 μg ml^−1^ Gm). *P. aeruginosa* strains were cultured in LB with antibiotics as required (50 μg ml^−1^ Gm). For *E. coli* whole cell analysis cells were grown in LB medium containing ampicillin overnight at 37 °C. For cell fractionation of *P. aeruginosa* strains carrying pJN105 cells were grown either in LB medium at 37 °C and arabinose (0.4%) was added after 4 hours of growth for 1 hour or in low inorganic phosphate medium containing 0.4% glucose at 30 °C until stationary phase (14 hours of growth) and arabinose (0.05%) was added at the beginning of the growth.

### Plasmid and strain construction

The plasmids and strains constructed in this work are shown in Table S2, primers are shown in Table S3. All amplifications were carried out with Q5 High Fidelity DNA polymerase (New England Biolabs), using PA14 genomic DNA as template. All constructs were sequenced to verify the absence of any mutation (GATC-biotech).

Amidase plasmids are derivatives of pssAmiA-AmiAH (based on the pT7.5 vector^60^) and were generated by PCR amplification of DNA encoding the N-terminal region of candidates using *P. aeruginosa* PA14 chromosomal DNA as template. Primers used to amplify sequences encoding signal peptide from each protein were designed to include the sequence between the start codon and the most likely signal peptidase cleavage site.

In pssAmiA-AmiAH derivatives, AmiA signal sequence was replaced by each candidate N-terminal sequence by enzymatic restriction using BamHI and XbaI or one-step sequence- and ligation-independent cloning (SLIC) as described in^61^. Each construct allows the expression of N-terminal genetic fusion to mature (signal peptide lacking) AmiA carrying a C-terminal hexa-histidine tag under the control of the *tatABC* constitutive promoter. Each of these clones carries the *tatA* ribosome-binding site, with identical spacing between the ribosome-binding site and the start codon.

pJNSphC_M-22_H (encoding a C-terminal hexa-histidine tag on SphC) was constructed by PCR amplification of *sphC* and a 97 bp region upstream of the annotated start codon including the putative missassigned methionine (M_−22_) and Shine Dalgarno sequence from *P. aeruginosa* strain PA14 in two steps. First, *sphC* was amplified using sphCSDup/sphCHisXbaIdown and cloned into pCR2.1 plasmid (TA cloning, Invitrogen). Six histidine codons followed by the stop codon were included in the reverse primer. Secondly, this construct was cloned into the *Eco*RI site of pJN105 under the transcriptional control of an arabinose-inducible promoter^62^. pJNSphC_M-22_H-KK was constructed by mutating the CGA and CGC codons encoding putative arginines respectively 16 and 15 amino acid upstream of the annotated start codon of *sphC* (R_−16_ and R_−15_) in Lysine by QuickChange site-directed mutagenesis (Stratagene) using sphCKKup/sphCKKdown and pCR2.1-SphC_M-22_H as template then cloning this construct into the *Eco*RI site of pJN105 under the transcriptional control of an arabinose-inducible promoter.

### Microscopy and outer membrane integrity assay

For phase-contrast microscopy, cells were grown to mid-exponential phase at 37 °C in LB medium and mounted on microscope slides covered with Poly-L-Lysine (Sigma-Aldrich) to immobilized cells. Fixed cells were imaged with a Zeiss AxioImager m2 equipped with a Hamamatsu OrcaR2 camera with a 100 × phase-contrast objective. The images were acquired with AxioVision software. For viability measurements, overnight cultures were adjusted to an OD_600_ of 1, serial dilutions from 10^−1^ to 10^−6^ were prepared in LB, and 5 µl of each dilution were spotted onto LB agar or LB agar + 2% (w/v) SDS. Plates were incubated overnight at 37 °C and photographed.

### Preparation of extracts, cell fractionation, protease accessibility experiments and analysis by SDS-PAGE and immunobloting

For *E. coli* whole cell extracts, cells (1 ml) were pelleted by centrifugation for 2 min at 13,000 × *g* at room temperature and resuspended in denaturing buffer (63 mM Tris-HCl pH 6.8, 10% glycerol, 2% SDS, 0.0025% bromophenol blue, 10% beta-mercaptoethanol). Fractionation of *E. coli* cells into spheroplasts (cytoplasm and membranes) and periplasmic fractions were done as described previously^63^ with slight modifications. Briefly, cells (20 ml) were pelleted by centrifugation for 10 min at 2,000 × *g*, and resuspended in 0.2 ml of spheroplast buffer (0.2 M Tris-HCl pH 8.0, 0.5 M sucrose and 0.5 M EDTA). A freshly prepared solution (10 μl) of lysozyme (2 mg/ml in water) was added, followed by the addition of 0.4 ml of spheroplast buffer in 0.4 ml of water. The cells were gently mixed and then incubated for 20 minutes at 4 °C. After 10 minutes the suspension was brought to 250 mM NaCl to facilitate the release of AmiAH as described before^64^. The spheroplasts were pelleted by centrifugation at 2,000 × *g* for 10 minutes and resuspended in 0.5 ml of spheroplast buffer 0.5X. The supernatant was reserved as the periplasmic fraction.

For protease accessibility experiments, the spheroplasts were incubated for 20 minutes on ice with a solution (13 μl) of Proteinase K (PK) (250 μg/ml) in the presence or the absence of 1% (v/v) Triton X-100 (TX). Next, PMSF, a Proteinase K inhibitor (2 mM) is added to the suspension. Samples (whole cells, spheroplasts, periplasm, spheroplasts treated with PK+/−TX) were precipitated with 12% TCA overnight at 4 °C and centrifuged at 13,000 × *g* for 30 minutes. Pellets were washed twice with 90% acetone (1 ml), air dried and resuspended in denaturing buffer.

*P. aeruginosa* whole cells, supernatant, spheroplasts and periplasmic fractions were prepared as described before^29,65^.

Protein samples derived from equivalent amounts of culture (*i.e*. optical density equivalents) were loaded in each lane and analyzed by SDS-PAGE and immunoblotting as described before^29^ using primary polyclonal antibodies directed against DsbA (kindly gifted by K.E. Jaeger – university of Heinrich-Heine, dilution 1:25000) and PlcH (kindly gifted by M.L. Vasil – University of Colorado, dilution 1:500), or monoclonal antibodies directed against EF-Tu (Hycult-biotech, dilution 1:20000), His_6_ epitope-tag (Penta His, Qiagen, dilution 1:1000) and Bla (Millipore 1:5000). For anti His_6_ detection, manufacturer instructions were followed. Peroxidase-conjugated anti-Mouse or anti-Rabbit IgGs (Sigma, dilution 1:5000) were used as secondary antibodies. Nitrocellulose membranes were developed with homemade enhanced chemiluminescence and exposed to X-ray film for the appropriate time or were scanned using ImageQuant LAS4000 analysis software (GE Healthcare Life sciences). Protein samples equivalent to 0.1 OD_600_ units were loaded for whole cell and spheroplasts analysis while protein samples equivalent to 0.2 OD_600_ units (anti-DsbA and anti-EF-Tu immunoblots) or 1 OD_600_ units (anti-His_6_ immunoblot) were used for periplasm analysis and protein samples equivalent to 1 OD_600_ units were used for supernatant analysis.

### Data availability

All data generated or analysed during this study are included in the published article (and its Supplementary Information files).

## Electronic supplementary material

Dataset 1

Dataset 2
