# Supplementary material for: Genome wide identification and experimental validation of Pseudomonas aeruginosa Tat substrates
Source: Sci Rep. 2018 Aug 9;8:11950. doi: 10.1038/s41598-018-30393-x (PMC6085387; doi:10.1038/s41598-018-30393-x)
Supplement: Supplementary file 1 — Dataset 1 [file 41598_2018_30393_MOESM1_ESM.pdf]

SUPPLEMENTARY INFORMATION

Genome wide identification and experimental validation of *Pseudomonas aeruginosa* Tat substrates

Maxime Rémi Gimenez[1], Govind Chandra[2], Perrine Van Overvelt[1], Romé Voulhoux[1], Sophie Bleves[1] and Bérengère Ize[1,*]

[1] Laboratoire d'Ingénierie des Systèmes Macromoléculaires (LISM-UMR7255), Institut de Microbiologie de la Méditerranée, CNRS and Aix-Marseille Univ. 31 Chemin Joseph Aiguier, CS 70071, 13402 Marseille cedex 09, France Marseille France.

[2] Department of Molecular Microbiology, John Innes Centre, Norwich Research Park, Norwich NR4 7UH, UK.

[*] Corresponding author. Tel: +33 (0) 491164487; Fax: +33 (0) 491712124. Email bize@imm.cnrs.fr

**Supplementary Figure S1**	**2**
**Supplementary Figure S2**	**3**
**Supplementary Figure S3**	**4**
**Supplementary Figure S4**	**5-10**
**Supplementary Table S2**	**11-12**
**Supplementary Table S3**	**13-14**
**Supplementary References**	**15**
**Supplementary File S1**	**16**

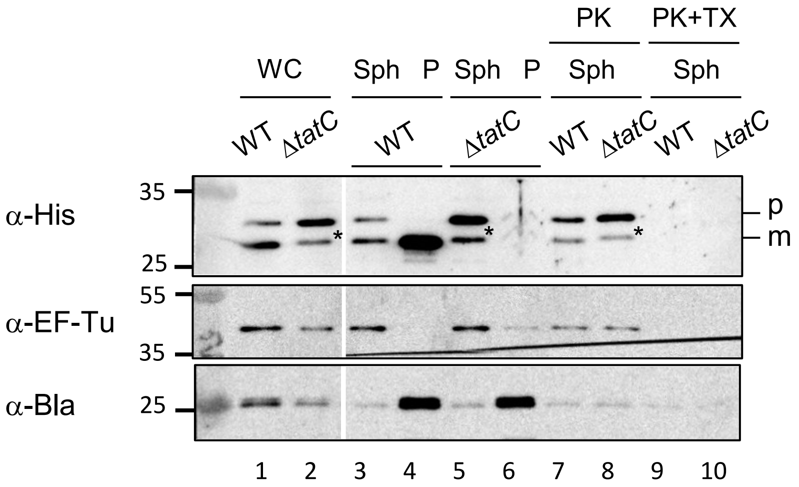

pssAmiA-AmiAH

Figure S1: AmiAH export to the periplasm is Tat-dependent

Immunoblot analysis of WT (MC4100) and Δ*tatC* (B1LK0) strains carrying pssAmiA-AmiAH (expressing *E. coli* AmiA with an His_6 epitope-tag) after cell fractionation and protease accessibility treatment. Whole cell extracts (WC), spheroplasts (Sph) and periplasm (P) treated or not with Proteinase K (PK) and Triton X-100 (TX) were loaded on a 12% SDS-PAGE gel. Blots were probed with anti-his tag (His_6), anti-elongation factor thermo unstable (EF-Tu) and anti-beta lactamase (Bla) antibodies. EF-Tu (43.3 kDa) is used as a cytoplasmic control and Bla (28.9 kDa) as a periplasmic control. The predicted sizes of ssAmiA-AmiAH unprocessed (precursor p) and processed (mature m) forms are 32.4 kDa and 28.6 kDa respectively and are indicated by arrows on the right of the gel. The form corresponding to AmiAH degradation product is indicated with an asterisk in the Δ*tatC* strain. The molecular masses (in kilodaltons) are indicated on the left of the gels.

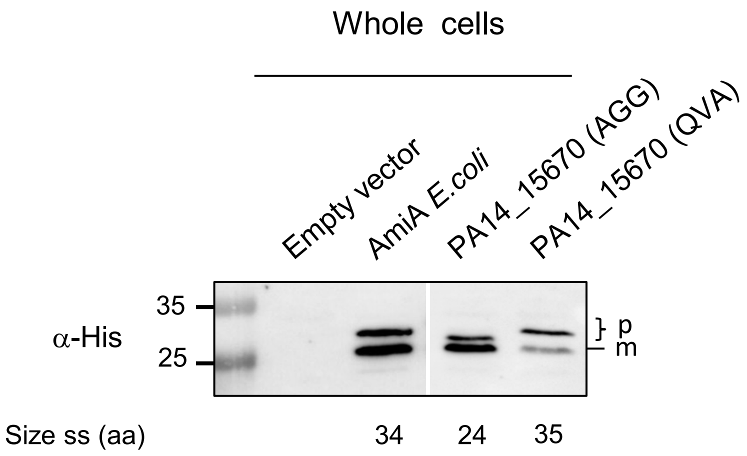

<u>Figure S2</u>: AmiAH export when fused to two versions of PA14_15670 signal peptide
Immunoblot analysis of *E. coli* Δ*ssamiAC* mutant (MCDSSAC) strain carrying pUNI-PROM (Empty vector), pssAmiA-AmiAH (expressing *E. coli* AmiA with an His_6 epitope-tag) or derivatives of pssAmiA-AmiAH where AmiA signal sequence has been replaced by two versions of PA14_15670 signal peptide (AmiAH is fused either after AGG or after QVA). Whole cell extracts were loaded on a 12% SDS-PAGE gel and blots were probed with anti-his tag (His_6). The predicted sizes of each signal peptide are indicated in amino acid at the bottom of the gel and the precursor (p) and mature (m) forms are indicated on the right of the gel. The molecular weight standards (in kilodaltons) are indicated on the left of the gels.

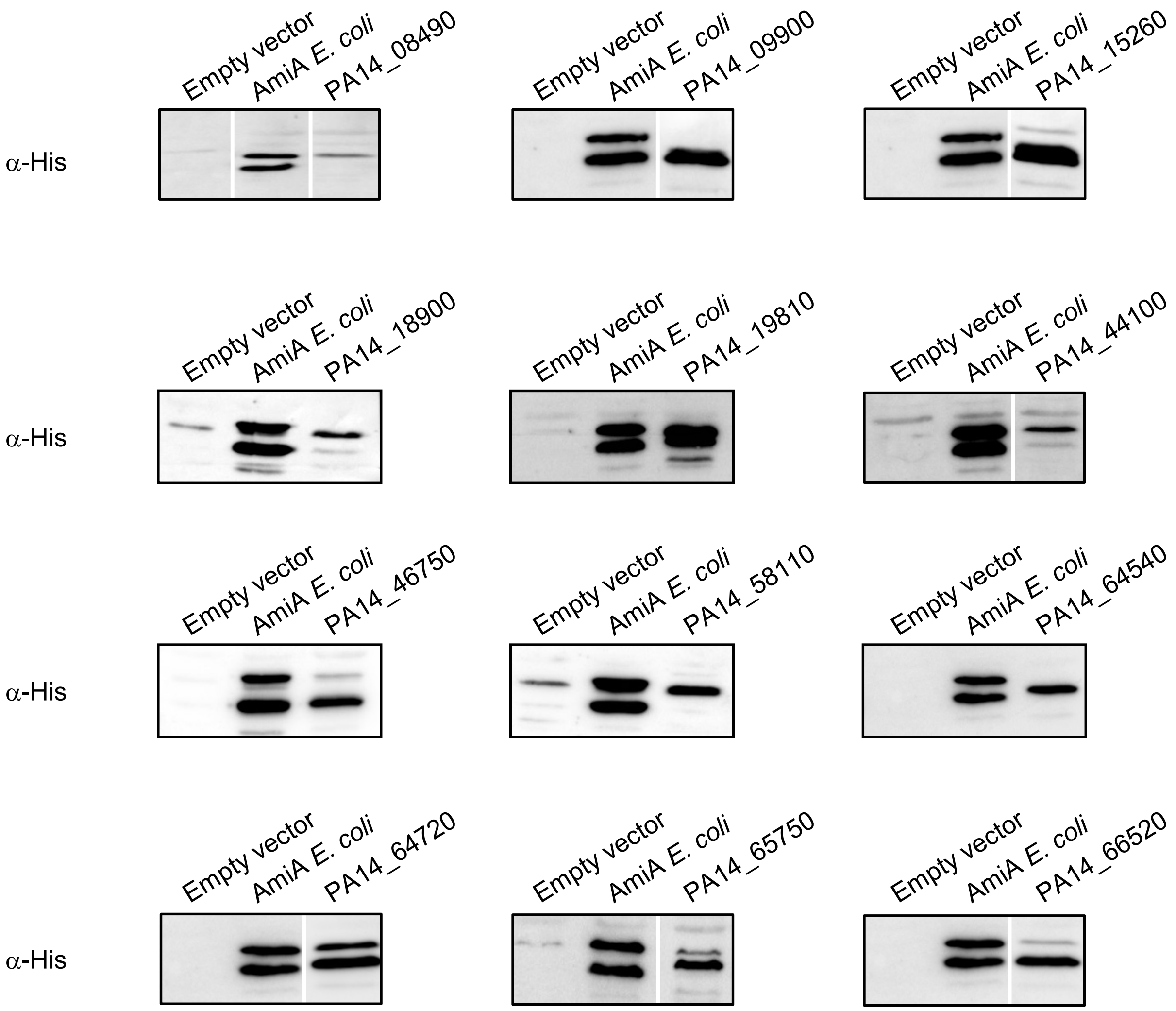

<u>Figure S3</u>: Production of reporter fusions not allowing Tat export
Immunoblot analysis of Δ*ssamiAC* mutant (MCDSSAC) carrying pUNI-PROM (empty vector), pssAmiA-AmiAH (expressing *E. coli* AmiA with an His_6 epitope-tag) or derivatives of pssAmiA-AmiAH where AmiA signal sequence has been replaced by various signal peptides. Whole cell extracts were loaded on a 12% SDS-PAGE gel and blots were probed with anti-His tag (His_6).

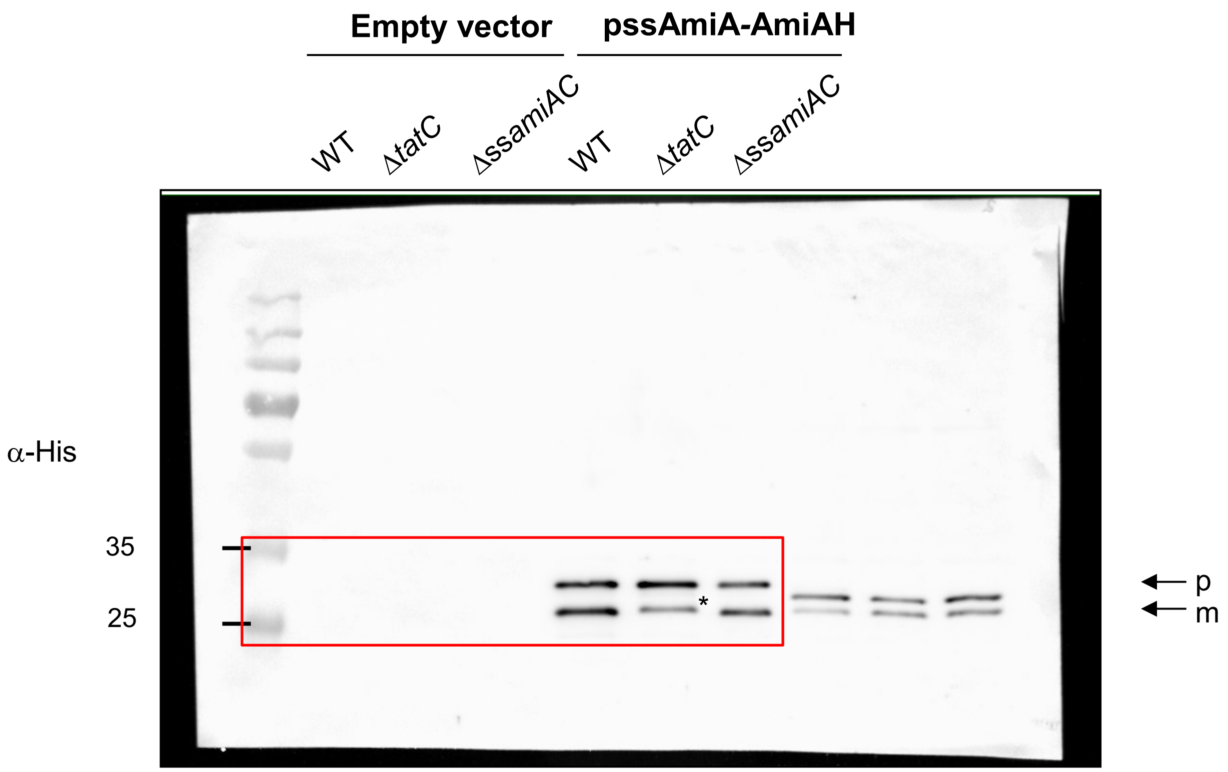

Full-lenght blot for Gimenez *et al*., Fig. 3C

α-His

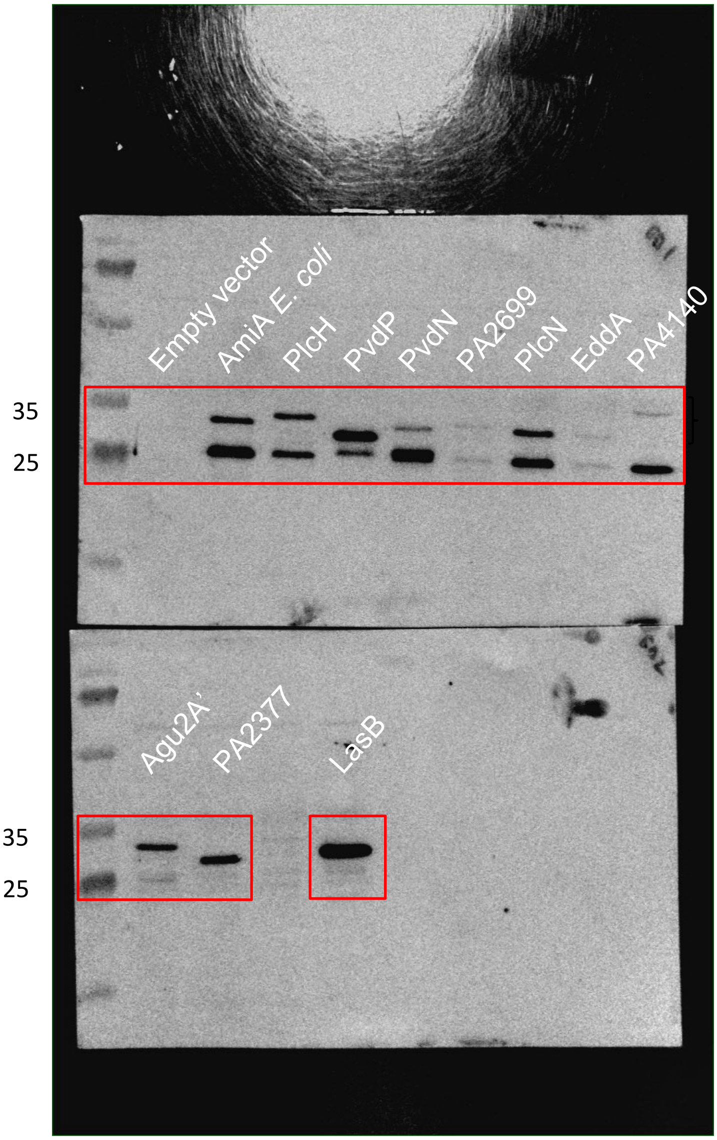

Full-lenght blot for Gimenez *et al*., Fig. 4C

A.

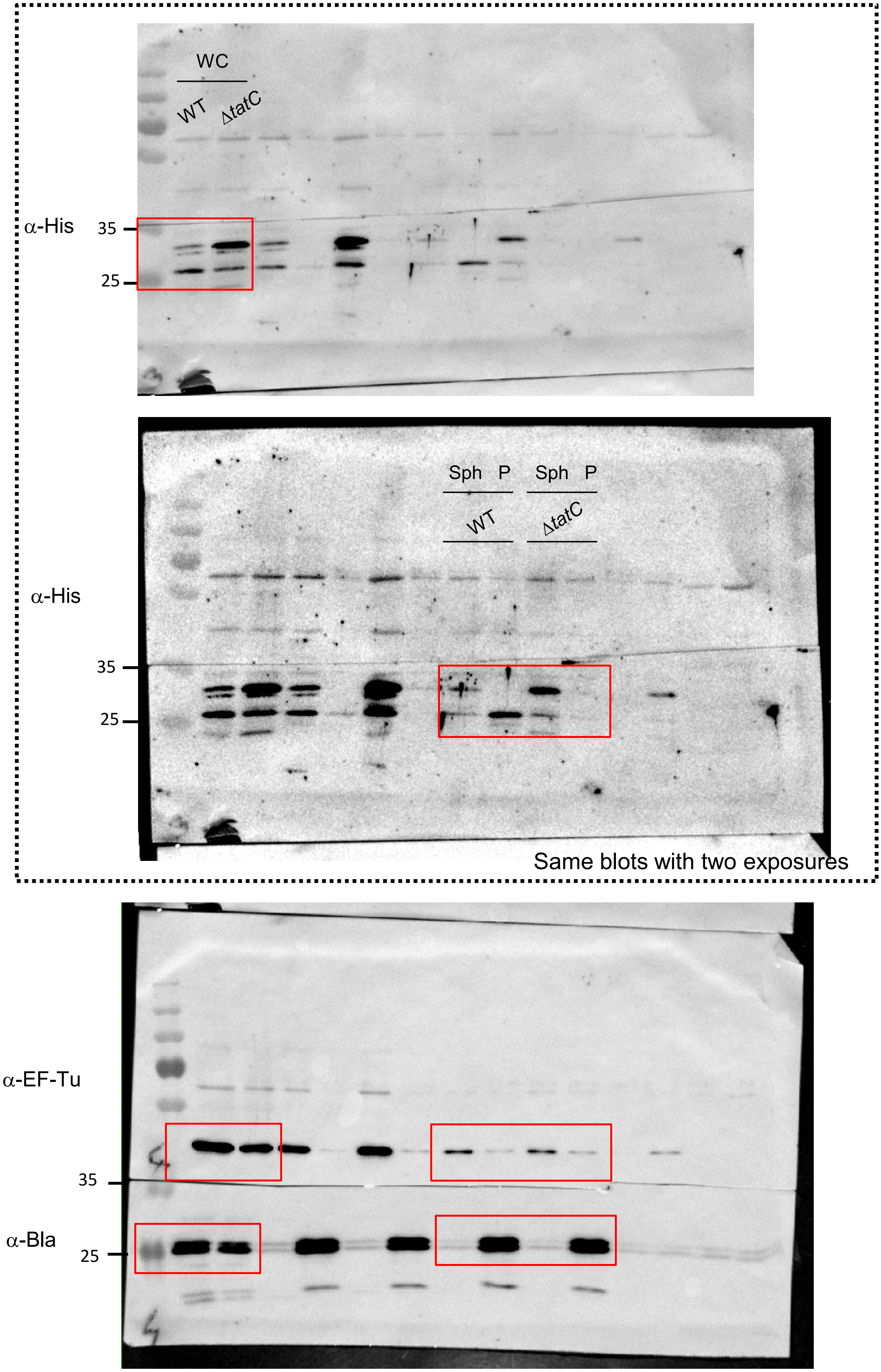

Full-lenght blots for Gimenez *et al.*, Fig. 5A

B.

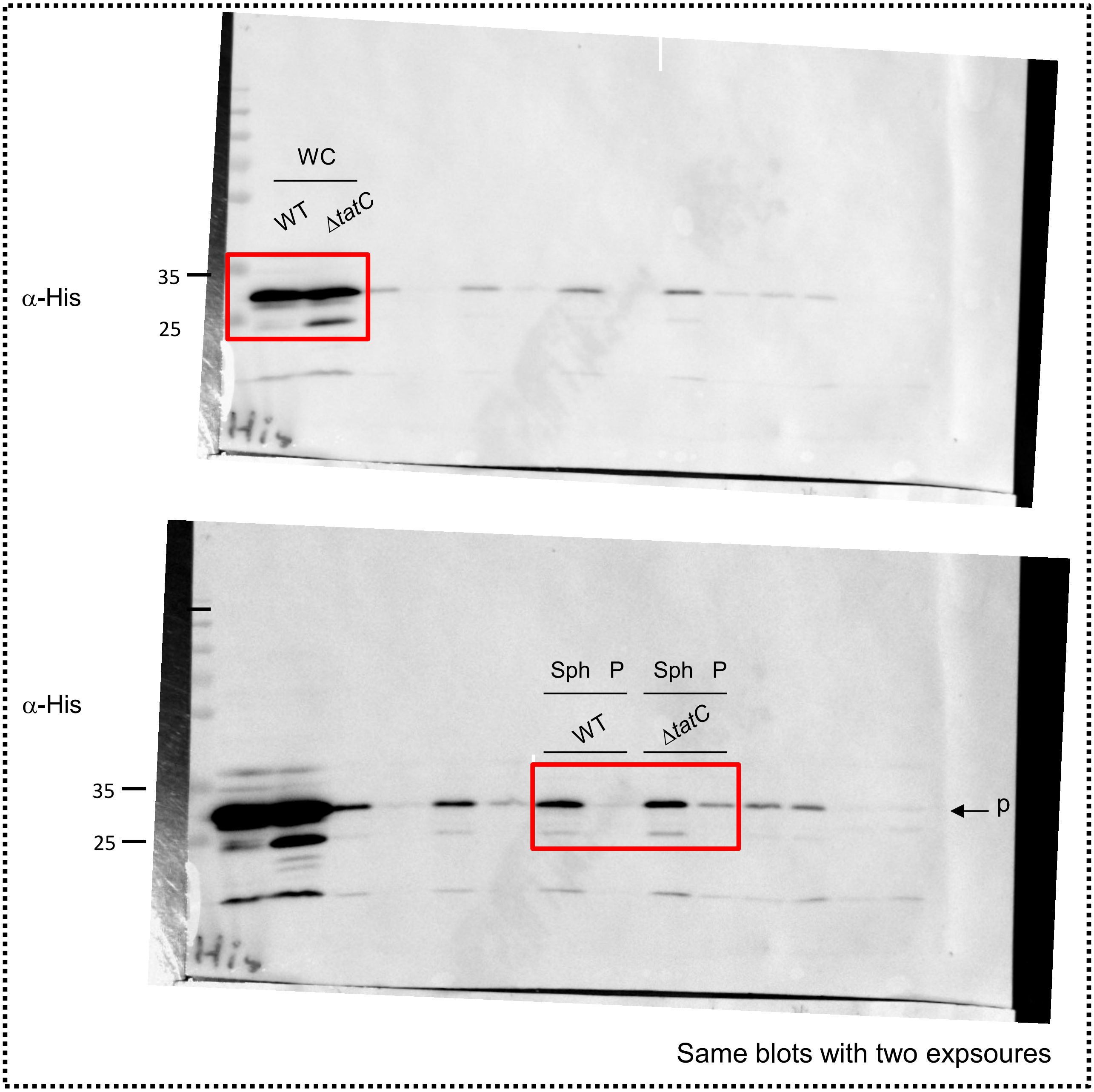

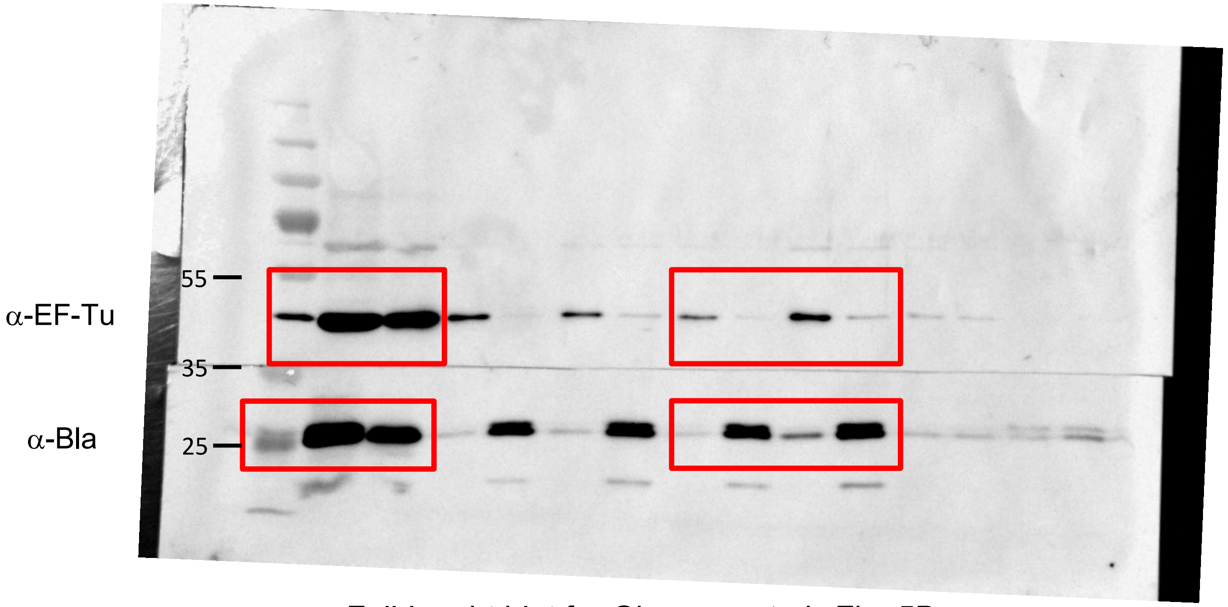

Full-lenght blot for Gimenez *et al*., Fig. 5B

C.

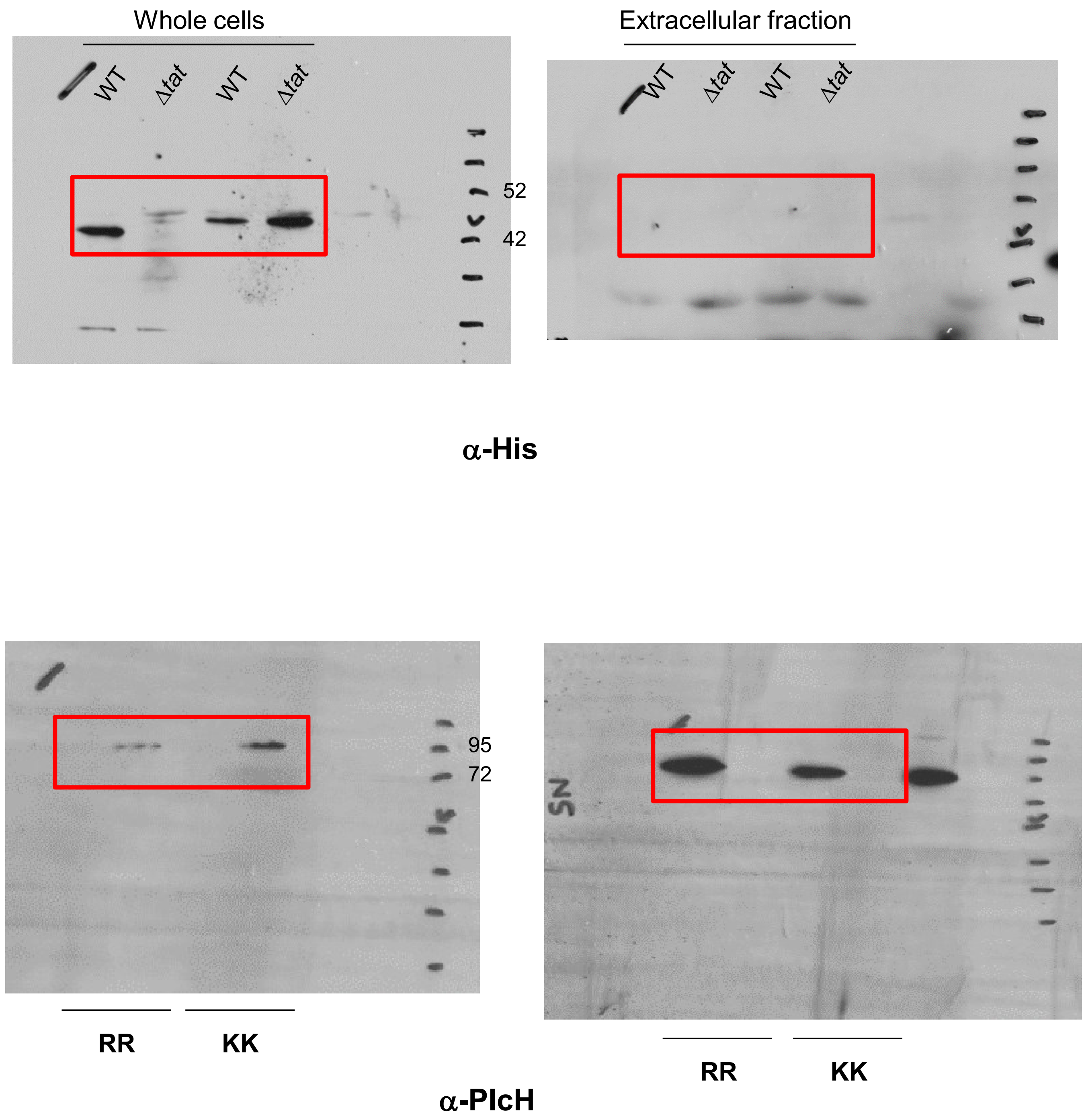

Full-lenght blot for Gimenez *et al*., Fig. 7C

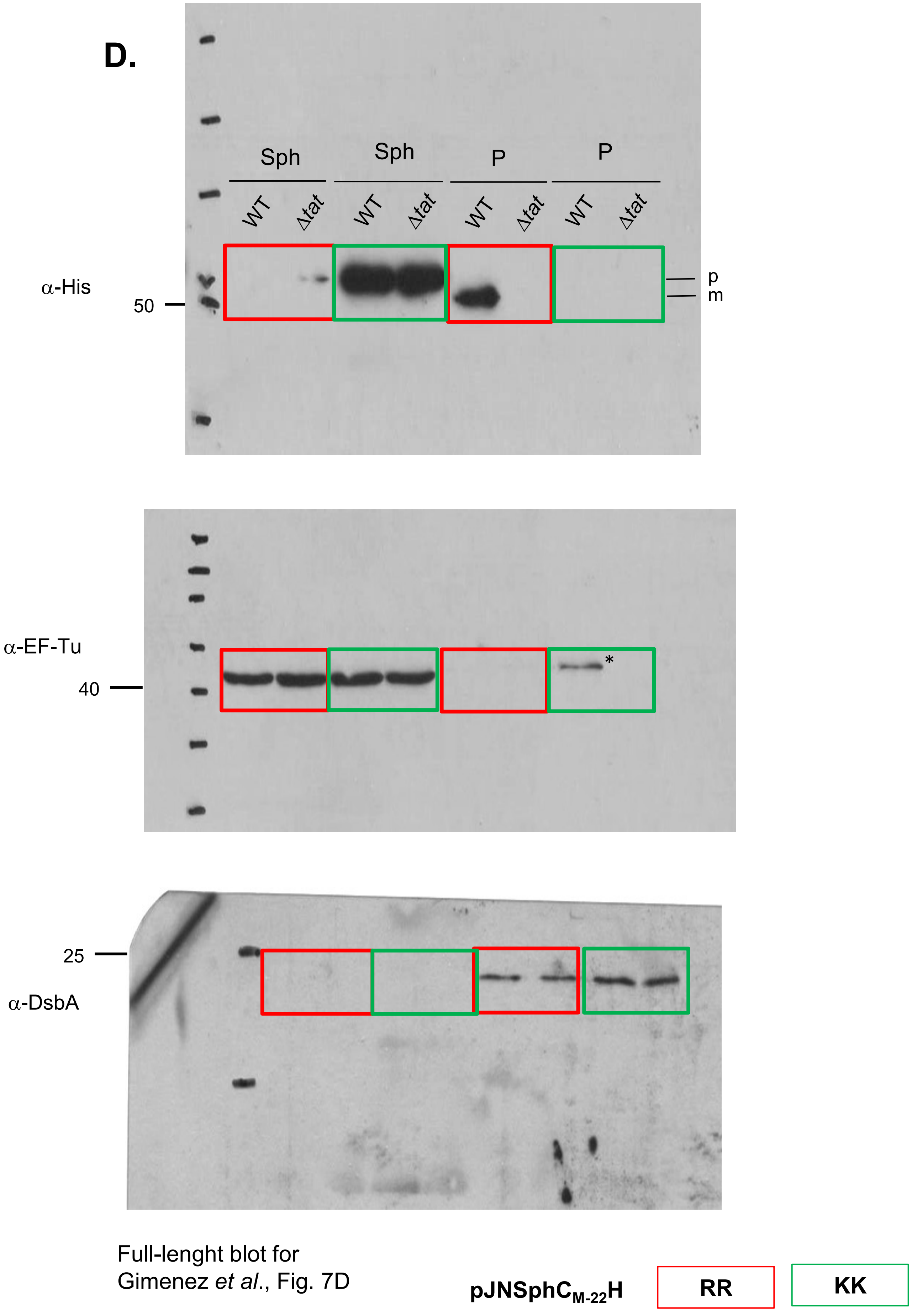

Full-lenght blot for
Gimenez *et al.*, Fig. 7D

Table S2. Strains and plasmids used in this study

**Bacterial Strains and plasmids**	**Description**	**Reference**
E. coli		
DH5α	F$^-$ Φ80*lac*ZΔM15 Δ(*lac*ZYA-*arg*F) U169 *rec*A1 *end*A1 *hsd*R17 (r_k^-, m_k^+) *pho*A *sup*E44 *thi*-1 *gyr*A96 *rel*A1 λ$^-$	Invitrogen
MC4100	F$^-$Δ*lacU169 araD139 rpsL150 relA1 ptsF rbs flbB5301*	(1)
B1LK0	MC4100 Δ*tatC*	(2)
MCDSSAC	as MC4100, *amiA*Δ2–33, *amiC*Δ2–32	(3)
P. aeruginosa		
PA14	Wild type	Laboratory collection
PA14Δ*tat*	*tatABC* deletion mutant	(4)
**Plasmids**		
pCR2.1	TA cloning, *lacZ*α, ColE1, f1 ori, Ap^R Km^R	Invitrogen
pCR2.1-SphC$_{M-22}$H	*PA14_70330*$_{His6}$ and 97bp promoter region cloned in pCR2.1	This work
pCR2.1-SphC$_{M-22}$H-KK	*PA14_70330*$_{His6}$ with a CGA>AAA, CGC>AAA substitutions in the 97bp promoter region cloned in pCR2.1.	This work
pRK2013	Km^R, ColE1, Tra+ Mob+	(5)
pJN105	*P. aeruginosa* expression vector with an arabinose inducible P$_{BAD}$ promoter; Gm^R	(6)
pJNSphC$_{M-22}$H	*PA14_70330*$_{His6}$ and 97bp promoter region cloned into EcoRI of pJN105	This work
pJNSphC$_{M-22}$H-KK	*PA14_70330*-$_{M-22}$H-KK and 97bp promoter region cloned into EcoRI of pJN105	This work
pUNI-PROM	pT7.5-derived vector allowing constitutive expression under the control of the *E. coli tat* promoter or inducible expression from the upstream T7 promoter (Ap^R)	(7)
pssAmiA-AmiAH	ssAmiA fused to mature AmiA carrying a C-terminal hexa-histidine tag in pUNI-PROM	(4)
pss01780-AmiAH	As pssAmiA-AmiAH but where AmiA signal peptide has been replaced by PA14_01780 signal peptide	This work
pss04790-AmiAH	As pssAmiA-AmiAH but where AmiA signal peptide has been replaced by PA14_04790 signal peptide	This work
pss08490-AmiAH	As pssAmiA-AmiAH but where AmiA signal peptide has been replaced by PA14_08490 signal peptide	This work
pss09900-AmiAH	As pssAmiA-AmiAH but where AmiA signal peptide has been replaced by PA14_09900 signal peptide	This work
pss10170-AmiAH	As pssAmiA-AmiAH but where AmiA signal peptide has been replaced by PA14_10170 signal peptide	This work
pss10370-AmiAH	As pssAmiA-AmiAH but where AmiA signal peptide has been replaced by PA14_10370 signal peptide	This work
pss13330-AmiAH	As pssAmiA-AmiAH but where AmiA signal peptide has been replaced by PA14_13330 signal peptide	This work
pss15260-AmiAH	As pssAmiA-AmiAH but where AmiA signal peptide has been replaced by PA14_15260 signal peptide	This work
pss15670AGG-AmiAH	As pssAmiA-AmiAH but where AmiA signal peptide has been replaced by PA14_15670 signal peptide up to Gly$_{24}$	This work
pss15670QVA-AmiAH	As pssAmiA-AmiAH but where AmiA signal peptide has been replaced by PA14_15670 signal peptide up to Ala$_{35}$	This work
pss16360-AmiAH	As pssAmiA-AmiAH but where AmiA signal peptide has been replaced by PA14_16360 signal peptide	This work
pss18900-AmiAH	As pssAmiA-AmiAH but where AmiA signal peptide has been replaced by PA14_18900 signal peptide	This work
pss19810-AmiAH	As pssAmiA-AmiAH but where AmiA signal peptide has been replaced by PA14_19810 signal peptide	This work
pss20200-AmiAH	As pssAmiA-AmiAH but where AmiA signal peptide has been replaced by PA14_20200 signal peptide	This work
pss21110-AmiAH	As pssAmiA-AmiAH but where AmiA signal peptide has been replaced by PA14_21110 signal peptide	This work
pss22560-AmiAH	As pssAmiA-AmiAH but where AmiA signal peptide has been replaced by PA14_22560 signal peptide	This work

pss30040-AmiAH	As pssAmiA-AmiAH but where AmiA signal peptide has been replaced by PA14_30040 signal peptide	This work
pss31820-AmiAH	As pssAmiA-AmiAH but where AmiA signal peptide has been replaced by PA14_31820 signal peptide	This work
pss33720-AmiAH	As pssAmiA-AmiAH but where AmiA signal peptide has been replaced by PA14_33720 signal peptide	This work
pss33740-AmiAH	As pssAmiA-AmiAH but where AmiA signal peptide has been replaced by PA14_33740 signal peptide	This work
pss33770-AmiAH	As pssAmiA-AmiAH but where AmiA signal peptide has been replaced by PA14_33770 signal peptide	This work
pss33900-AmiAH	As pssAmiA-AmiAH but where AmiA signal peptide has been replaced by PA14_33900 signal peptide	This work
pss34510-AmiAH	As pssAmiA-AmiAH but where AmiA signal peptide has been replaced by PA14_34510 signal peptide	This work
pss35300-AmiAH	As pssAmiA-AmiAH but where AmiA signal peptide has been replaced by PA14_35300 signal peptide	This work
pss37100-AmiAH	As pssAmiA-AmiAH but where AmiA signal peptide has been replaced by PA14_37100 signal peptide	This work
pss37790-AmiAH	As pssAmiA-AmiAH but where AmiA signal peptide has been replaced by PA14_37790 signal peptide	This work
pss40200-AmiAH	As pssAmiA-AmiAH but where AmiA signal peptide has been replaced by PA14_40200 signal peptide	This work
pss43790-AmiAH	As pssAmiA-AmiAH but where AmiA signal peptide has been replaced by PA14_43790 signal peptide	This work
pss44100-AmiAH	As pssAmiA-AmiAH but where AmiA signal peptide has been replaced by PA14_44100 signal peptide	This work
pss46750-AmiAH	As pssAmiA-AmiAH but where AmiA signal peptide has been replaced by PA14_46750 signal peptide	This work
pss48450-AmiAH	As pssAmiA-AmiAH but where AmiA signal peptide has been replaced by PA14_48450 signal peptide	This work
pss49250-AmiAH	As pssAmiA-AmiAH but where AmiA signal peptide has been replaced by PA14_49250 signal peptide	This work
pss53360-AmiAH	As pssAmiA-AmiAH but where AmiA signal peptide has been replaced by PA14_53360 signal peptide	This work
pss54770-AmiAH	As pssAmiA-AmiAH but where AmiA signal peptide has been replaced by PA14_54770 signal peptide	This work
pss57570-AmiAH	As pssAmiA-AmiAH but where AmiA signal peptide has been replaced by PA14_57570 signal peptide	This work
pss58110-AmiAH	As pssAmiA-AmiAH but where AmiA signal peptide has been replaced by PA14_58110 signal peptide	This work
pss61150-AmiAH	As pssAmiA-AmiAH but where AmiA signal peptide has been replaced by PA14_61150 signal peptide	This work
pss62110-AmiAH	As pssAmiA-AmiAH but where AmiA signal peptide has been replaced by PA14_62110 signal peptide	This work
pss63605-AmiAH	As pssAmiA-AmiAH but where AmiA signal peptide has been replaced by PA14_63605 signal peptide	This work
pss64270-AmiAH	As pssAmiA-AmiAH but where AmiA signal peptide has been replaced by PA14_64270 signal peptide	This work
pss64540-AmiAH	As pssAmiA-AmiAH but where AmiA signal peptide has been replaced by PA14_64540 signal peptide	This work
pss64720-AmiAH	As pssAmiA-AmiAH but where AmiA signal peptide has been replaced by PA14_64720 signal peptide	This work
pss65750-AmiAH	As pssAmiA-AmiAH but where AmiA signal peptide has been replaced by PA14_65750 signal peptide	This work
pss66520-AmiAH	As pssAmiA-AmiAH but where AmiA signal peptide has been replaced by PA14_66520 signal peptide	This work
pssSphC$_{M-22}$-AmiAH	As pssAmiA-AmiAH but where AmiA signal peptide has been replaced by PA14_70330 signal peptide	This work
pss73040-AmiAH	As pssAmiA-AmiAH but where AmiA signal peptide has been replaced by PA14_73040 signal peptide	This work

Table S3. Primer sequences used in this study

Primer name	Primer Sequence
**pJNSphC-$_{M-22}$H and derivative**	
SphCSDup	5'- CCGCAGCGCAGGACCGATAGGGGA-3'
SphCHisXbaIdown	5'- TCTAGATCAGTGATGGTGATGGTGATGGGTCACGCCCAGGATGGAAGA-3'
SphCKKup	5'-CAAGTCATCTGTCGAAAAAACAGCTCCTGCAACG-3'
SphCKKdown	5'-CGTTGCAGGAGCTGTTTTTTCGACAGATGACTTG-3'
**pssAmiA-AmiAH and derivatives**	
AmiAEcFor	5'-TCTAGAAAAGACGAACTTTTA-3'
T7.5Rev2	5'-TGATTTAATTCTCATGTTTGA-3'
AmiAR	5'-GCGCAAGCTTTTAGTGATGGTGATGGTGATGTCGCTTTTTCGAATGTGC-3'
T7	5'-GTAATACGACTCACTATAGGGC-3'
01780ssFor	5'-ATCTACCACAGAGGAGGATCCATGAGCCGATCCAAC-3'
01780ssRev	5'-TAAAAGTTCGTCTTTTCTAGAGGCGCGTACGCCCGGCAG-3'
04790ssFor	5'-ATCTACCACAGAGGAGGATCCATGAGCGACACCACT-3'
04790ssRev	5'-TAAAAGTTCGTCTTTTCTAGACGCGGTGGCGAGGAAGGC -3'
08490ssFor	5'-ATCTACCACAGAGGAGGATCCATGTCCGGCTGGGAA-3'
08490ssRev	5'-TAAAAGTTCGTCTTTTCTAGAGGCGTCCGCCCCGGTCGAC-3'
09900ssFor	5'-ATCTACCACAGAGGAGGATCCATGCATAAGAGAACG-3'
09900ssRev	5'-TAAAAGTTCGTCTTTTCTAGAGGCCGAGGCCTGGCTCGC-3'
10170ssFor	5'-ATCTACCACAGAGGAGGATCCATGCCGACCCGCCGC-3'
10170ssRev	5'-TAAAAGTTCGTCTTTTCTAGAGGCCGCGGCGAGGGTGGC-3'
10370ssFor	5'-AAAGGATCCATGCACGACCCCATCCAGC-3'
10370ssRev	5'-TAAAAGTTCGTCTTTTCTAGAGGCTTCGGCGGGCTGGAT-3'
13330ssFor	5'-AAAGGATCCATGAGTGGGATGGACCTC-3'
13330ssRev	5'-TAAAAGTTCGTCTTTTCTAGAGGCGATCACCGCCGGCGC-3'
15260ssFor	5'-ATCTACCACAGAGGAGGATCCATGACCCGACGTACC-3'
15260ssRev	5'-TAAAAGTTCGTCTTTTCTAGAGGCGTGTCCGGCGGCGGC-3'
15670ssFor	5'-ATCTACCACAGAGGAGGATCCATGACATTTACCCGT-3'
15670ssRev-AGG	5'-TAAAAGTTCGTCTTTTCTAGAGCCGCCGGCGCCGAGGCC-3'
15670ssRev-QVA	5'-TAAAAGTTCGTCTTTTCTAGACTGCGCCACCTGCGGACG-3'
16360ssFor	5'-ATCTACCACAGAGGAGGATCCATGACCATCTCCCGC-3'
16360ssRev	5'-TAAAAGTTCGTCTTTTCTAGAAGCGCGGAGGATCTCGGC-3'
18900ssFor	5'-ATCTACCACAGAGGAGGATCCATGGACGCCGCGACT-3'
18900ssRev	5'-TAAAAGTTCGTCTTTTCTAGACACCAGTGCTACCAGGCC-3'
19810ssFor	5'-CGGGATCCATGACAACAACAAAAAGGC-3'
19810ssRev	5'-TAAAAGTTCGTCTTTTCTAGAGGCCGAGGCCTGGGTGGC-3'
20200ssFor	5'-ATCTACCACAGAGGAGGATCCATGAGCGACGACACG-3'
20200ssRev	5'-TAAAAGTTCGTCTTTTCTAGAGGCCCGGGCCTCGCGGCC-3'
21110ssFor	5'-ATCTACCACAGAGGAGGATCCATGATTTCGAAAAGC-3'
21110ssRev	5'-TAAAAGTTCGTCTTTTCTAGACGCCTGGATGCTCGACGG-3'
22560ssFor	5'-ATCTACCACAGAGGAGGATCCATGAATCGACGTTCC-3'
22560ssRev	5'-TAAAAGTTCGTCTTTTCTAGAGGCGCTGGCCAGCTTGCC-3'
30040 ssFor	5'-CGGGATCCATGAGCCTGGAGAAAAAAG-3'
30040ssRev	5'-TAAAAGTTCGTCTTTTCTAGAGGCGCTGGCGGGGAGCGCGA-3'
31820ssFor	5'-ATCTACCACAGAGGAGGATCCATGCCCGCTTTGAGT-3'
31820ssRev	5'-TAAAAGTTCGTCTTTTCTAGAGGCGAAGGCTCGCGGGCT-3'
33720ssFor	5'-CGGGATCCATGAACGACCGTCGTACCT-3'
33720ssRev	5'-TAAAAGTTCGTCTTTTCTAGAGGCGCGGAGCGACTGGGC-3'
33740ssFor	5'-ATCTACCACAGAGGAGGATCCATGACGGTTTCTCGA-3'
33740ssRev	5'-TAAAAGTTCGTCTTTTCTAGAAGCCACCGGCAGCGCCGC-3'
33770ssFor	5'-ATCTACCACAGAGGAGGATCCATGAGACGAACCCGT-3'
33770ssRev	5'-TAAAAGTTCGTCTTTTCTAGAGGCCAGGGCGATCAGGGG-3'
33900ssFor	5'-ATCTACCACAGAGGAGGATCCATGAAGCGCTCGTTC-3'
33900ssRev	5'-TAAAAGTTCGTCTTTTCTAGAGGCCAGGGCGTCGCGCCA-3'
34510ssFor	5'-CGGGATCCATGTGCCTGGACGACCCGA-3'
34510ssRev	5'-TAAAAGTTCGTCTTTTCTAGAGGCACGCGCCTGCAGGCT-3'

35300ssFor	5’-ATCTACCACAGAGGAGGATCCATGCCCGATGACAAG-3’
35300ssRev	5’-TAAAAGTTCGTCTTTTCTAGAGGCGGGCGTCTGCATGGC-3’
37100ssFor	5’-ATCTACCACAGAGGAGGATCCATGCACCAACCCGAA-3’
37100ssRev	5’-TAAAAGTTCGTCTTTTCTAGAGGCGCTGGCCACGCCGGG-3’
37790ssFor	5’-ATCTACCACAGAGGAGGATCCATGCACAGAACTTCG-3’
37790ssRev	5’-TAAAAGTTCGTCTTTTCTAGAGGCCCAGGCCGGGGCGCGCC-3’
40200ssFor	5’-ATCTACCACAGAGGAGGATCCATGAACAGCAAGATC-3’
40200ssRev	5’-TAAAAGTTCGTCTTTTCTAGAGGCGAGGGCGCGCCGGGC-3’
43790ssFor	5’-CGGGATCCATGAGCCTGGCCAATCCCT-3’
43790ssRev	5’-TAAAAGTTCGTCTTTTCTAGAGGCGAGGGCGAGTAGCGG-3’
44100ssFor	5’-ATCTACCACAGAGGAGGATCCATGCGGTTGCACAAG-3’
44100ssRev	5’-TAAAAGTTCGTCTTTTCTAGAAGCCGGCCCCCGCGACGC-3’
46750ssFor	5’-ATCTACCACAGAGGAGGATCCATGAGCGAGCGACTC-3’
46750ssRev	5’-TAAAAGTTCGTCTTTTCTAGAGCCGAGGAAGTGGCTGGC-3’
48450ssFor	5’-ATCTACCACAGAGGAGGATCCATGACCCGCAGACAT-3’
48450ssRev	5’-TAAAAGTTCGTCTTTTCTAGATGCCGCCGCCTGTCCACG-3’
49250ssFor	5’-CGGGATCCATGAACCTCACCCGTCGTG-3’
49250ssRev	5’-TAAAAGTTCGTCTTTTCTAGAGGCGCGGACCAGGATCGG-3’
53360ssFor	5’-CGGGATCCATGACCGAAAACTGGAAAT-3’
53360ssRev	5’-TAAAAGTTCGTCTTTTCTAGAGGCCAGGGCGCGCCGGAG-3’
54770ssFor	5’-ATCTACCACAGAGGAGGATCCATGAATCGCAACCGT-3’
54770ssRev	5’-TAAAAGTTCGTCTTTTCTAGAGGACCGGTTCCAGGCGAC-3’
57570ssFor	5’-ATCTACCACAGAGGAGGATCCATGAGTAATGACGGC-3’
57570ssRev	5’-TAAAAGTTCGTCTTTTCTAGAAGCCTTGGCTTTGGCACT-3’
58110ssFor	5’-ATCTACCACAGAGGAGGATCCATGCCGTCGCTGTAT-3’
58110ssRev	5’-TAAAAGTTCGTCTTTTCTAGAGGCGGGCGCTTCGTTCGG-3’
61150ssFor	5’-ATCTACCACAGAGGAGGATCCATGTCCAACCGTGATATATC-3’
61150ssRev	5’-TAAAAGTTCGTCTTTTCTAGAAGCGAACGCCTGGCTGCC-3’
62110ssFor	5’-ATCTACCACAGAGGAGGATCCATGCTGATCAAGATTCCCT-3’
62110ssRev	5’-TAAAAGTTCGTCTTTTCTAGAGGCCAGCGCCAATCCGGC-3’
63605ssFor	5’-ATCTACCACAGAGGAGGATCCATGGATATGAACCGT-3’
63605ssRev	5’-TAAAAGTTCGTCTTTTCTAGAGGCGAAGGCCTCCGTGGG-3’
64270ssFor	5’-ATCTACCACAGAGGAGGATCCATGAAACGTCGCAGTCTGC-3’
64270ssRev	5’-TAAAAGTTCGTCTTTTCTAGAGGCCTGGATGGACCAGGA-3’
64720ssFor	5’-ATCTACCACAGAGGAGGATCCATGAAAATCCGTCGT-3’
64720ssRev	5’-TAAAAGTTCGTCTTTTCTAGAAGCTTGCGCGGCAAGACA-3’
64540ssFor	5’-ATCTACCACAGAGGAGGATCCATGAAGGGGCCGGAG-3’
64540ssRev	5’-TAAAAGTTCGTCTTTTCTAGACTGTAGCCGGCACCCCGA-3’
65750ssFor	5’-ATCTACCACAGAGGAGGATCCATGCTGCGCAGACTC-3’
65750ssRev	5’-TAAAAGTTCGTCTTTTCTAGAGGCCCAGGCGACACCGGT-3’
66520ssFor	5’-ATCTACCACAGAGGAGGATCCATGCATCCCTGGGCA-3’
66520ssRev	5’-TAAAAGTTCGTCTTTTCTAGAGGCCAGCGTGCGCGCCGC-3’
70330$_{M-22}$ssFor	5’-ATCTACCACAGAGGAGGATCCATGCCAAGTCATCTGTCGC-3’
70330ssRev	5’-TAAAAGTTCGTCTTTTCTAGACGCCAGCGCGGGGTTTCC-3’
73040ssFor	5’-ATCTACCACAGAGGAGGATCCATGAAGCGCCGTCGCCT-3’
73040ssRev	5’-TAAAAGTTCGTCTTTTCTAGAGGCGCTGGCGGCCAGGAA-3’

Supplementary References

1. Casadaban, M. J. & Cohen, S. N. Lactose genes fused to exogenous promoters in one step using a Mu-lac bacteriophage: in vivo probe for transcriptional control sequences. *Proc. Natl. Acad. Sci. U. S. A.* **76,** 4530–3 (1979).

2. Bogsch, E. G. *et al.* An essential component of a novel bacterial protein export system with homologues in plastids and mitochondria. *J. Biol. Chem.* **273,** 18003–18006 (1998).

3. Ize, B., Stanley, N. R., Buchanan, G. & Palmer, T. Role of the *Escherichia coli* Tat pathway in outer membrane integrity. *Mol. Microbiol.* **48,** 1183–1193 (2003).

4. Ball, G. *et al.* Contribution of the Twin Arginine Translocation system to the exoproteome of *Pseudomonas aeruginosa*. *Sci. Rep.* **6,** (2016).

5. Figurski, D. H. & Helinski, D. R. Replication of an origin-containing derivative of plasmid RK2 dependent on a plasmid function provided *in trans*. *Proc. Natl. Acad. Sci.* **76,** 1648–1652 (1979).

6. Newman, J. R. & Fuqua, C. Broad-host-range expression vectors that carry the L-arabinose-inducible *Escherichia coli araBAD* promoter and the *araC* regulator. *Gene* **227,** 197–203 (1999).

7. Jack, R. L. *et al.* Coordinating assembly and export of complex bacterial proteins. *EMBO J.* **23,** 3962–3972 (2004).

```
#!/usr/bin/perl
use strict;
use Bio::SeqIO;
use Carp;

# {{{ POD

=head1 Name

jic_tatfind_fasta.pl

=head1 Description

This is a reimplementation of the TATFIND script version 1.4
the original version which was described in
https://doi.org/10.1046/j.1365-2958.2002.03090.x

The following modifications were made.

=over 2

=item 1.
The TAT motif is not hard-coded to RR.

=item 2.
There is no restriction on the format of the input fasta file.
TATFIND requires that the first line of the input protein sequences
contain
at least 60 amino acids. There is no such restriction in this
script.

=item 3.
This script relies on BioPerl modules to be installed and
available.

=back

=head1 Example

 perl jic_tatfind_fasta.pl -infile pa14.faa

=head1 Options

=over 2

=item -infile

Input file to read. This should be a protein fasta format file.
This has to be provided.

=item -outfile

If specified, this is the file output will be written to. Otherwise
output is to the terminal (STDOUT). The output is in three tab-
```

```
separated
columns.

 1. Protein identifier
 2. The first 60 (or whatever is the value of $howfarin)
 amino acids of the protein
 3. Calculated phobicity of the hydrophobic region.

=item -fasta

If specified, protein sequences predicted to be TAT substrates
are written out to this file in the fasta format.

=back

Below is some description of each of the subroutines.

=cut
# }}}

# {{{ Getopt::Long stuff
use Getopt::Long;
my $infile;
my $ofn;
my $outfas;
GetOptions (
"infile=s" => \$infile,
"outfile:s" => \$ofn,
"fasta:s" => \$outfas
);

# }}}

# {{{ Open the outfile. Defaults to STDOUT.
my $ofh;
if($ofn) {
  open($ofh, ">", $ofn);
}
else {
  open($ofh, ">&STDOUT");
}
select($ofh);
# }}}

# Some file globals
my $howfarin=60;
my $hydrolen=13;
my $pr_phob;
my $pr_seq;
my $tat='(RK|KR|RR)';
# my $tat='RR';
# Below, the length over which hydrophobic region is looked for.
my $extent_len=22;
```

```
# {{{ %phob # A hash containing numbers for phobicity calculation.

my %phob=(
A => 0.02,
R => -0.42,
N => -0.77,
D => -1.04,
C => 0.77,
Q => -1.10,
E => -1.14,
G => -0.80,
H => 0.26,
I => 1.81,
L => 1.14,
K => -0.41,
M => 1.00,
F => 1.35,
P => -0.09,
S => -0.97,
T => -0.77,
W => 1.71,
Y => 1.11,
V => 1.13
);

# }}}


unless($infile) {
if($ARGV[0]) { $infile = $ARGV[0]; }
}
unless(-r $infile and -s $infile) {
my $croak_msg = qq(\nAn input filename has to be provided\n);
$croak_msg .= qq(and the file should be readable.\n);
$croak_msg .= qq(\nTry perldoc $0 to get some help.\n);
print($croak_msg, "\n");
exit;
}


my $seqout;
my $faah;
if($outfas) {
  open($faah, ">", $outfas);
  $seqout=Bio::SeqIO->new(-fh => $faah, -format => 'fasta');
}

# {{{ Go thorough all the sequences in the input fasta file.
my $seqio=Bio::SeqIO->new('-file' => $infile);
while(my $seqobj=$seqio->next_seq()) {
      my $aa_seq = $seqobj->seq();
      my $locus_tag = $seqobj->display_name();

print(STDERR "$locus_tag\r");
```

```
#print(STDERR "$aa_seq\n");
if(&rule1($aa_seq) and &rule2($aa_seq) and (&rule3a($aa_seq)
  or &rule3b($aa_seq) or &rule4($aa_seq))) {
  print("$locus_tag\t$pr_seq\t$pr_phob\n");
  if(ref($seqout)) {
    $seqout->write_seq($seqobj);
  }
}
#last;    ### debugging only
}
# }}}

# close file handles and exit.
close($ofh);
if(defined($faah)) {
close($faah);
}
exit;


# {{{ rule1

=head2 Sub rule1

Arguments: aa_seq

Returns: boolean

 if (substr($aa_seq, 0, $howfarin)=~/([HAPKRNTGSDQE]RR[APKRNTGSDQE]
[IWFLVYMCHAPNT][ILVMF])/) {

=cut

sub rule1 {
#  warn("Rule 1");
  my $aa_seq = uc(shift(@_));
  if (substr($aa_seq, 0, $howfarin)=~m/[HAPKRNTGSDQE]
$tat[APKRNTGSDQE][IWFLVYMCHAPNT][ILVMF]/) {
    return(1);
  }
}

# }}}

# {{{ rule2

=head2 Sub rule2

Arguments: aa_seq

Returns: boolean

Rule2 is applied to the 22 amino acids following the twin arginine.
Checks to see if there is a hydrophobic stretch of at least
```

```
$hydrolen (13)
residues in the 22 amino acids following the RR.

 $extent=~m/RR(.{22})/g
 =~m/[^DERK]{$hydrolen}/

=cut

sub rule2 {
#  warn("Rule 2");
  my $aa_seq=shift(@_);
  my $extent=substr($aa_seq, 0, $howfarin);
  my @tocheck = $extent=~m/$tat(.{$extent_len})/g;
  if(grep {$_=~m/[^DERK]{$hydrolen}/} @tocheck) {
    return(1);
  }
  else {
    return(0);
  }
}

# }}}

# {{{ rule3a

=head2 Sub rule3a

Arguments: aa_seq

Returns: boolean

 if (substr($aa_seq, 0, $howfarin)=~/RR[DERK]/)

=cut

sub rule3a {
#  warn("Rule 3a");
  my $aa_seq = uc(shift(@_));
  my $extent = substr($aa_seq, 0, $howfarin);
    if ($extent=~/$tat[DERK]/) {
      my $phob = &phobicity($aa_seq);
      if($phob < 8.0) {
        $pr_phob = sprintf("%.3f", $phob);
        $pr_seq = $extent;
        return(1);
      }
    }
    else {return(0);}
}

# }}}

# {{{ rule3b
```

```
=head2 Sub rule3b

Arguments: aa_seq

Returns: boolean

 if (substr($aa_seq, 0, $howfarin)=~/RR.{3}[DERK]/)

=cut

sub rule3b {
#  warn("Rule 3b");
  my $aa_seq = uc(shift(@_));
  my $extent = substr($aa_seq, 0, $howfarin);
    if ($extent=~/$tat.{3}[DERK]/) {
      my $phob = &phobicity($aa_seq);
      if($phob < 8.0) {
        $pr_phob = sprintf("%.3f", $phob);
        $pr_seq = $extent;
        return(1);
      }
    }
    else {return(0);}
}

# }}}

# {{{ rule4

=head2 Sub rule4

Arguments: aa_seq

Returns: boolean

The rule is a basic residue immediately preceeding the hydrophobic
region.

 if(grep {$_=~m/[KR][^DERK]{$hydrolen,}/} @tocheck)

=cut


sub rule4 {
#  warn("Rule 4");
  my $aa_seq=shift(@_);
  my $extent=substr($aa_seq, 0, $howfarin);
  my @tocheck = $extent=~m/$tat(.{$extent_len})/g;
  if(grep {$_=~m/[KR][^DERK]{$hydrolen,}/} @tocheck
      or grep {$_=~m/^[^DERK]{$hydrolen,}/} @tocheck
    ) {
    my $phob = &phobicity($aa_seq);
    if($phob < 8.0) {
      $pr_phob = sprintf("%.3f", $phob);
```

```
      $pr_seq = $extent;
      return(1);
    }
  }
  else {
    return(0);
  }
}

# }}}

# {{{ phobicity

=head2 Sub phobicity

Arguments: aa_seq

Returns: minphobicity

Uses the global configuration variables $howfarin and $hydrolen

=cut

sub phobicity {
#  warn("phobicity");
  my $aa_seq=shift(@_);
  my $extent=substr($aa_seq, 0, $howfarin);
  my @tocheck = $extent=~m/$tat(.{$extent_len})/g;
#  &print_list(\@tocheck);   ### debugging only
  my $minphobicity=999999999;
  foreach my $tc (@tocheck) {
    my $outpos=0;
    while (my $hydro = substr($tc, $outpos, $hydrolen)) {
      if(length($hydro) < $hydrolen) {last;}
      my $score=0;
      my $pos = 0;
      while(my $aa=substr($hydro, $pos, 1)) {
        $score+=$phob{$aa};
        $pos+=1;
      }
      if($score < $minphobicity) {$minphobicity = $score;}
      $outpos+=1;
    }
  }
  return($minphobicity);
}
# }}} end of sub phobicity #####

# {{{ print_list (diagnostics only).
sub print_list {
  my $lr=shift(@_);
  my @list=@{$lr};
  foreach my $el (@list) {
    print(STDERR "$el\n");
```

```
  }
}
# }}}

=head1 Author

Govind Chandra E<lt>govind.chandra@jic.ac.ukE<gt>


=cut

__END__
```
